# Inhibition of Heat Shock Protein 90β by Catalpol: A Potential Therapeutic Approach for Alleviating Inflammation‐Induced Cartilage Injuries in Osteoarthritis

**DOI:** 10.1002/advs.202503909

**Published:** 2025-04-25

**Authors:** Zhenwei Zhou, Binghua Zhang, Lang Liu, Jie Yang, Yuting Wang, Cheng Lv, He Zhang, Yuchi Wei, Zhanliang Jiang, Zeyu Peng, Daqing Zhao, Xiangyang Leng, Xiangyan Li, Hang Su, Haisi Dong

**Affiliations:** ^1^ Affiliated Hospital of Changchun University of Traditional Chinese Medicine Changchun University of Chinese Medicine Changchun Jilin Province 130000 China; ^2^ College of Traditional Chinese Medicine Changchun University of Chinese Medicine Changchun Jilin Province 130000 China; ^3^ Northeast Asia Institute Research of Traditional Chinese Medicine Changchun University of Chinese Medicine Changchun Jilin Province 130000 China

**Keywords:** bMSN, Catalpol, chondrocyte metabolism, Hsp90β, osteoarthritis

## Abstract

Osteoarthritis (OA) is a degenerative joint disease characterized by the metabolic dysfunction of chondrocytes. A promising therapeutic strategy for OA involves suppressing the catabolism of the chondrocyte and promoting its anabolism to restore joint homeostasis. Here, it is demonstrated that Catalpol, a natural compound, can promote chondrocyte anabolic and proliferation, while inhibiting the catabolic activities and oxidative stress, thereby maintaining the dynamic balance of the extracellular matrix and alleviating inflammation‐induced cartilage damage. Mechanistically, it has been discovered that Catalpol acts as a direct inhibitor of heat shock protein 90β (Hsp90β), and the amino acids ASP88, THR179, ASP49, and ASN46 of N‐terminal domain‐Hsp90β are confirmed as the binding sites for Catalpol. Knockdown of Hsp90β in primary chondrocytes demonstrates a similar biological effect as Catalpol treatment. Moreover, to develop a nanoparticle‐based interventional platform for OA management, biodegradable mesoporous silica nanoparticles (bMSN) are prepared to load Catalpol (Ca‐bMSN). The engineered Ca‐bMSN is able to penetrate into the chondrocytes, prolong retention in the joint space, and mitigate OA progression. These findings shed light on a potential mechanism by which Catalpol modulates chondrocyte metabolism, offering a promising therapeutic strategy for OA treatment.

## Graphical abstract

The schematic diagram delineates the chondroprotective effects of Ca‐bMSN in OA models. The upper portion of the diagram depicts the synthesis process of Ca‐bMSN. The lower section illustrates the post‐injection of Ca‐bMSN in joint cavity, catalpol released and subsequently mediates the reduction of oxidative stress, ECM catabolism, apoptosis, and inflammation, while promoting ECM anabolism, thereby contributing to cartilage protection.

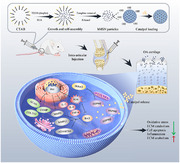

## 1 Introduction

Osteoarthritis (OA), a degenerative joint disease characterized by cartilage breakdown, affects an estimated 250 million people worldwide. Notably, a staggering 80% of individuals over the age of 75 in China are reported to be diagnosed with OA.^[^ ^1^ ^]^ The typical clinical features of OA include joint pain, swelling, stiffness, and limited range of motion of joint, significantly impacting patient quality of life. As a degenerative disease, OA is usually caused by ageing, chronic strain, and trauma. The excessive wear of articular cartilage surfaces induced by chronic strain was the most common predisposing risk factor for OA. Nonsteroidal anti‐inflammatory drugs (NSAIDs) are currently the mainstay of treatment for OA. However, they have not been shown to stop cartilage degeneration or promote its repair.^[^ ^2^ ^]^ In addition, their use is often linked to significant kidney and heart problems. Therefore, there is a critical need for a safe and effective treatment that can slow the progression of OA.

Chondrocytes represent the sole cellular component within the articular cartilage, enclosed by an extracellular matrix (ECM) abundant in proteoglycans and type II collagen to maintain joint function.^[^ ^3^ ^]^ The excessive degradation contributes to the breakdown of the ECM is a hallmark feature of OA. Chondrocytes are responsible for maintaining cartilage health through a delicate balance of synthesis and degradation of the ECM, achieved by regulating anabolic and catabolic activities.^[^ ^4^ ^]^ Anabolic processes primarily focus on the synthesis of ECM components by upregulating cartilage anabolic factors like COL2A1 and ACAN in chondrocytes. Conversely, the catabolic phenotype is characterized by the abnormal production of ECM‐degrading enzymes, such as matrix metalloproteinase 3, 9, 13 (Mmp3, Mmp9, Mmp13) and a disintegrin and metalloproteinase with thrombospondin motifs 4, 5 (Adamts4, Adamts5), by chondrocytes. During OA progression, pro‐inflammatory cytokines like interleukin‐1β (IL‐1β) and tumor necrosis factor‐α (TNF‐α) emerge as key players. These mediators induce chondrocyte dedifferentiation and promote oxidative stress, ultimately disrupting the balance between anabolic and catabolic processes. This imbalance can lead to chondrocyte apoptosis, further accelerating cartilage degeneration.^[^ ^5^ ^]^ Therefore, therapeutic strategies that enhance chondrocyte anabolic activity while inhibiting catabolic processes hold significant promise for managing OA.

Catalpol, an iridoid glycoside derived from the root of *Rehmannia*, exhibits significant therapeutic potential for various diseases due to its anti‐inflammatory, antioxidant, and anti‐apoptotic properties.^[^ ^6^ ^]^ An increasing body of evidence suggests that Catalpol significantly reduces Mmp3, Mmp9, and Adamts5 and increases mitochondrial biogenesis, suggesting a potential anti‐cartilage degradation effect in ATDC5 cells and rat chondrocytes.^[^ ^7^, ^8^, ^9^ ^]^ However, these studies have only focused on the effects of Catalpol on the breakdown of cartilage in chondrocytes. Further confirmation is needed to determine whether Catalpol can also promote cartilage growth. In addition, the exact mechanism and target of Catalpol in OA treatment remain unclear.

The intra‐articular (IA) injection of small molecule drugs is an effective way to treat knee OA. However, this method suffers from rapid clearance from the joint cavity due to interaction with synovial fluid constituents, significantly reducing therapeutic efficacy. To address this, drug‐delivery systems were developed. Mesoporous silica nanoparticles (MSN) are promising drug delivery carriers due to their adjustable pore size, high porosity, excellent biocompatibility, and convenient drug loading.^[^ ^10^ ^]^ MSNs protect drugs from premature degradation before release and provide sustained release, enhancing therapeutic effect and reducing side effects.^[^ ^11^ ^]^ However, the skeleton structure of “─Si─O─Si─” is stable, and it may be extremely concentrated in organs of body, such as liver, spleen and bladder, which may cause a serious problem such as inflammatory reaction, oxidative damage, and organ fibrosis. Biodegradable MSN (bMSN) not only retains the advantage of drug release performance, but also can be degraded rapidly in vivo, which can avoid the accumulation of cytotoxicity. Although studies have shown that bMSNs may have the potential to be used as drug delivery vehicles of drugs for OA therapy, the evidence of their effectiveness in the OA model is still lacking.^[^ ^12^ ^]^

In this study, bMSN was synthesized to load Catalpol (Ca‐bMSN). The effects of Catalpol, bMSN, and Ca‐bMSN on chondrocytes were then investigated in both OA chondrocytes and OA animal models. In addition, the molecular targets and underlying mechanism of Catalpol on regulating OA were investigated by a series of experiments such as drug affinity responsive target stability (DARTS), cellular thermal shift assay (CETSA), isothermal titration calorimetry (ITC), molecular dynamics (MD), and small interfering RNAs (siRNAs) assays. This study offered the definitive mechanism for the cartilage protective effects of Catalpol and provided a promising combinatory approach for OA.

## 2 Results

### 2.1 Catalpol Regulates Chondrocyte Proliferation and Metabolism

**Figure 1:**
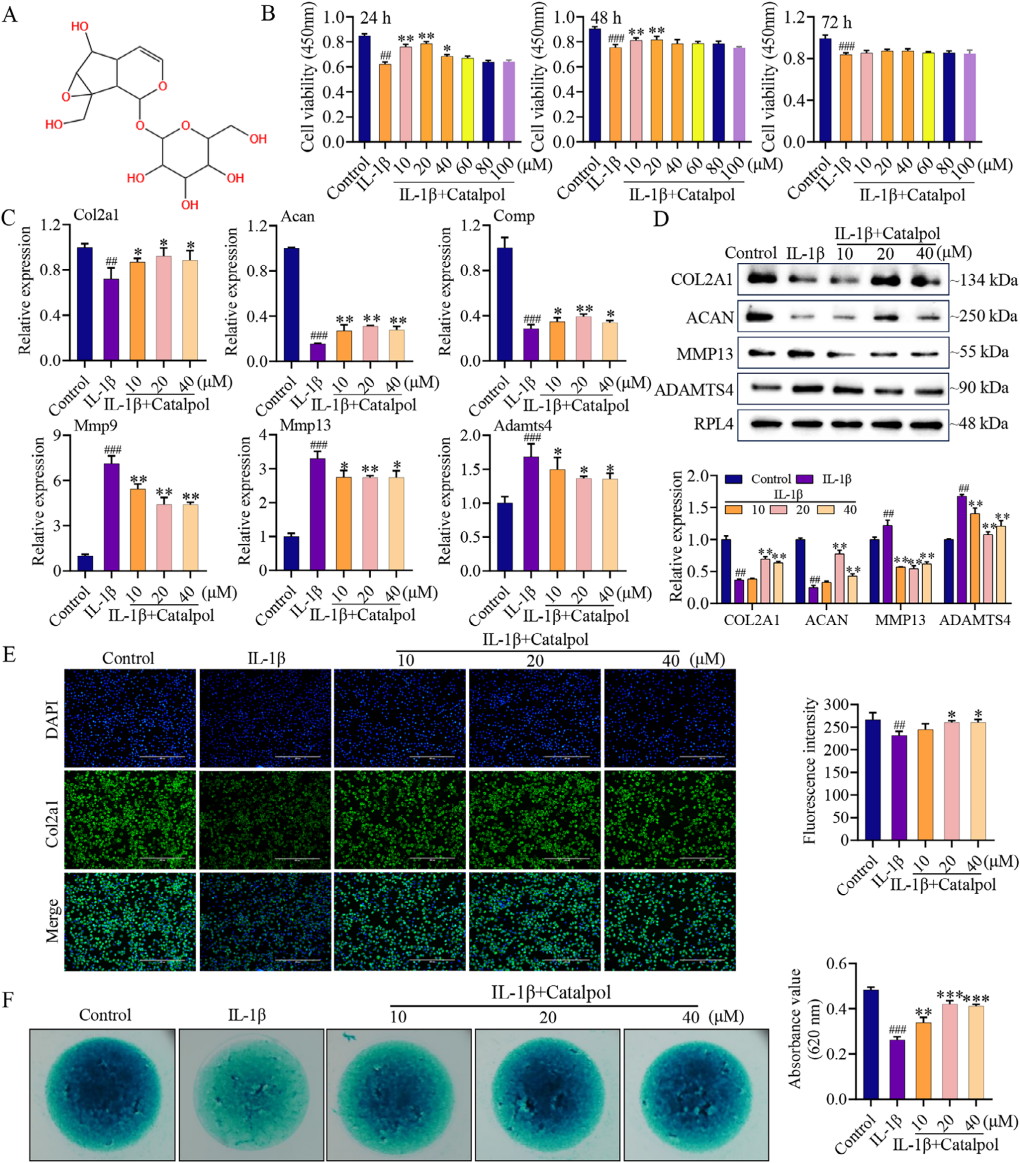
Catalpol promotes anabolism in primary chondrocytes and inhibits catabolism. A) Structure of Catalpol. B) The effect of different concentrations Catalpol on IL‐1β induced primary chondrocytes viability was detected by CCK‐8 assay at 24, 48, and 72 h, respectively (*n* = 5, *p < 0.05 and **p < 0.01). C) qRT‐PCR analysis of the expression level of anabolism marker genes (*Col2a1, Acan, Comp*) and catabolism marker genes (*Mmp9, Mmp13, Adamts4*) in chondrocytes treated with IL‐1β or IL‐1β combined with different concentrations of Catalpol at 24 h (*n* = 3, *p < 0.05, ## or **p < 0.01, ###*p* < 0.001 when compared with control group and IL‐1β group, respectively). D) After treatment with IL‐1β or IL‐1β and combined with different concentrations of Catalpol for 24 h, the expression of COL2A1, ACAN, MMP13, and ADAMTS4 in the primary chondrocytes was analyzed using Western blot assay. The quantification result is shown at the bottom (*n* = 3, ## or **p < 0.01 when compared with control group and IL‐1β group, respectively). E) After treatment with different concentrations of Catalpol for 24 h, the expression of COL2A1 in the primary chondrocytes was analyzed using immunofluorescence assay. The quantification result is shown at right. Scale bar = 400 µm (*n* = 3, *p < 0.05, ##*p* < 0.001 when compared with control group and IL‐1β group, respectively). F) The level of acidic polysaccharides in the primary chondrocytes under the treatment with different concentrations of Catalpol for day 7 were analyzed by Alcian blue staining. The quantification result is shown at right (*n* = 3, *p < 0.05, ##*p* < 0.01, ***p < 0.001 when compared with control group and IL‐1β group, respectively). Data are shown as mean ± SEM. *P*‐values were obtained by one‐way ANOVA with multiple comparisons.

Chondrocytes, the only cell type residing in cartilage tissue, play a crucial role in regulating ECM homeostasis. Therefore, we initially assessed the influence of Catalpol (**Figure** **1**A) on the proliferation of IL‐1β induced primary mouse chondrocytes at 24, 48, and 72 h using varying Catalpol concentrations. As shown in Figure 1B, Catalpol significantly promoted chondrocyte proliferation, with the most effective concentration being 20 × 10^−6^ m at 24 and 48 h. We further evaluated the effect of Catalpol on chondrocyte anabolism and catabolism. In an OA cell model induced by 10 ng mL^−1^ IL‐1β, Catalpol at concentrations of 10, 20, and 40 × 10^−6^ m effectively stimulated the expression of anabolic marker genes (*Col2a1*, *Acan*, *Comp*) (Figure 1C). Conversely, Catalpol reduced the expression of catabolic marker genes (*Mmp9*, *Mmp13*, *Adamts4*) (Figure 1C). This effect was confirmed at the protein level, with Catalpol increasing COL2A1 and ACAN expression while decreasing MMP13 and ADAMTS4 (Figure 1D,E). Similar results were observed in human chondrocytes (Figure S1A,B, Supporting Information). Alcian blue staining indicated that Catalpol treatment improved the expression of acidic polysaccharides in IL‐1β‐induced OA chondrocytes compared to the control group (Figure 1F). These findings collectively suggest that Catalpol exerts a protective effect on chondrocytes by facilitating their proliferation and anabolic activity while inhibiting their catabolic processes.

### 2.2 Anti‑oxidative Effects of Catalpol on the Osteoarthritic Chondrocytes

**Figure 2:**
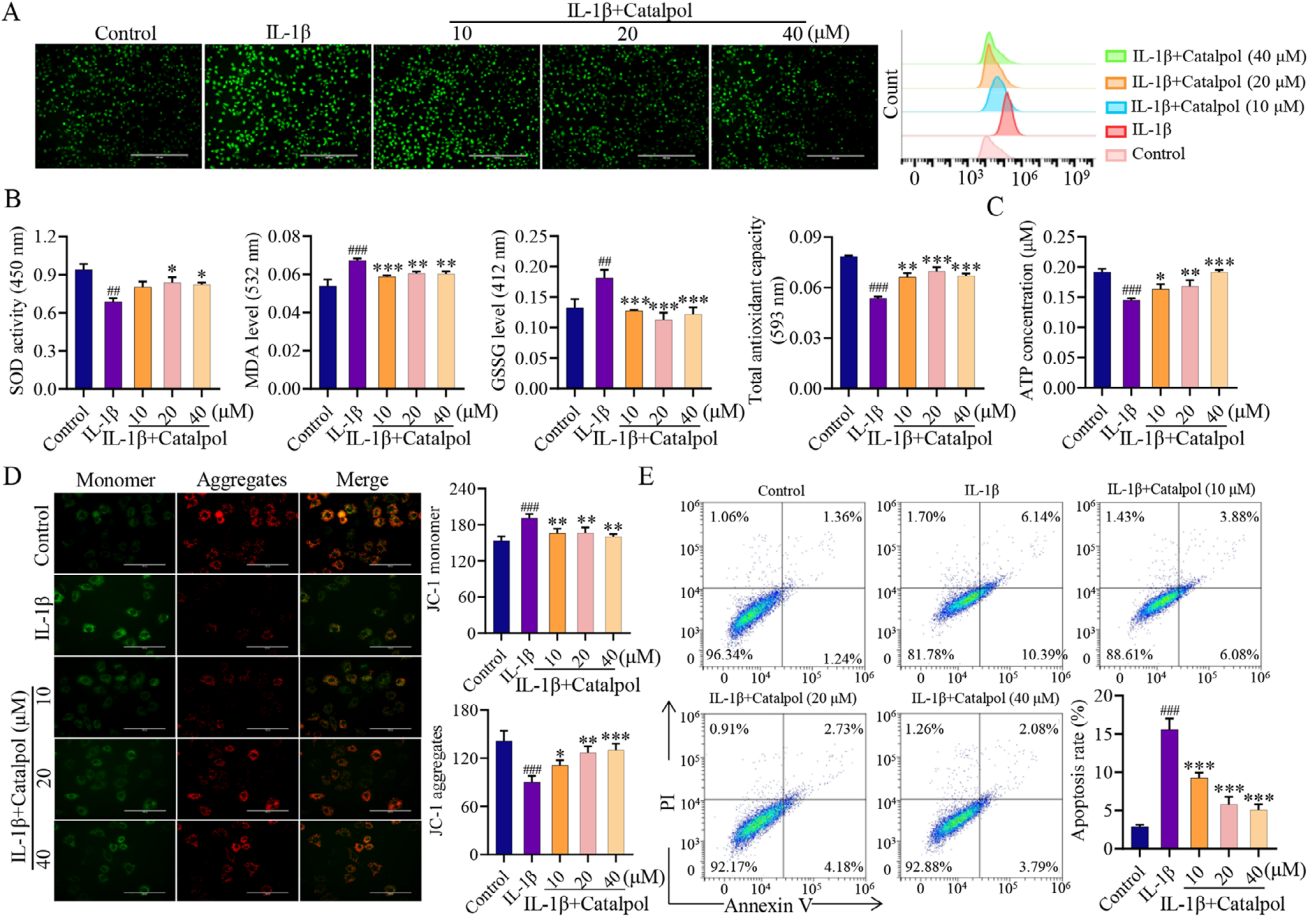
Protective effect of Catalpol on chondrocytes against oxidative stress injury induced by IL‐1β. A) ROS production in chondrocytes was assessed through the utilization of the ROS probe DCFH‐DA under the treatment with IL‐1β or different concentrations of Catalpol. Fluorescence microscopy was used to capture images, while flow cytometric analysis was conducted to quantify the results. Scale bar = 400 µm. B) The level of SOD, MDA, GSSG, and total antioxidant capacity in chondrocytes after treated with IL‐1β or different concentrations of Catalpol were detected using specific kits (*n* = 3, *p < 0.05, ## or **p < 0.01, ### or ***p < 0.001 when compared with control group and IL‐1β group, respectively). C) The concentration of ATP in the in chondrocytes under the treatment with IL‐1β or different concentrations of Catalpol was detected by ATP assay kit (*n* = 3, *p < 0.05, **p < 0.01, ### or ***p < 0.001 when compared with control group and IL‐1β group, respectively). D) Mitochondrial membrane potential. Red, JC‐1 aggregates; green, JC‐1 monomer. Scale bar = 100 µm (*n* = 3, *p < 0.05, **p < 0.01, ### or ***p < 0.001 when compared with control group and IL‐1β group, respectively). E) Cell apoptosis was detected by flow cytometry under the treatment with IL‐1β or different concentrations of Catalpol (*n* = 3, ### or ***p < 0.001 when compared with control group and IL‐1β group, respectively). Data are shown as mean ± SEM. *P*‐values were obtained by one‐way ANOVA with multiple comparisons.

Oxidative stress and apoptosis are the key factors in the decrease of chondrocytes and the loss of cartilage. Therefore, we investigated whether Catalpol possesses anti‐oxidative effects in OA cells. As shown in **Figure** **2**A,B, Catalpol treatment significantly reduced excessive reactive oxygen species (ROS), malondialdehyde (MDA), and glutathione disulfide (GSSG) levels in the OA cell model at concentrations of 20 × 10^−6^ and 40 × 10^−6^ m, while significantly increasing the level of antioxidant enzyme superoxide dismutase (SOD). Notably, Catalpol displayed the most significant increase in total antioxidant capacity at 20 × 10^−6^ m. Similarly, Catalpol inhibited IL‐1β‐induced ROS increase in human chondrocytes (Figure S2, Supporting Information). In addition, Catalpol treatment maintained mitochondrial membrane potential and increased ATP production compared to the untreated OA cell model (Figure 2C,D). We further investigated the influence of Catalpol (10, 20, 40 × 10^−6^ m) on OA cell apoptosis. Flow cytometric analysis results revealed that Catalpol reversed IL‐1β‐induced apoptosis (16.53%) in a dose‐dependent manner, with reduced percentages at 10 × 10^−6^ m (9.96%), 20 × 10^−6^ m (6.91%), and 40 × 10^−6^ m (5.87%) (Figure 2E). Collectively, these results indicate that Catalpol has the ability to suppress the generation of ROS and decrease chondrocyte apoptosis, thereby enhancing chondrocyte proliferation, anabolism and ultimately promoting cartilage injury repair.

### 2.3 Preparation and Physicochemical Characterization of Ca‐bMSN

**Figure 3:**
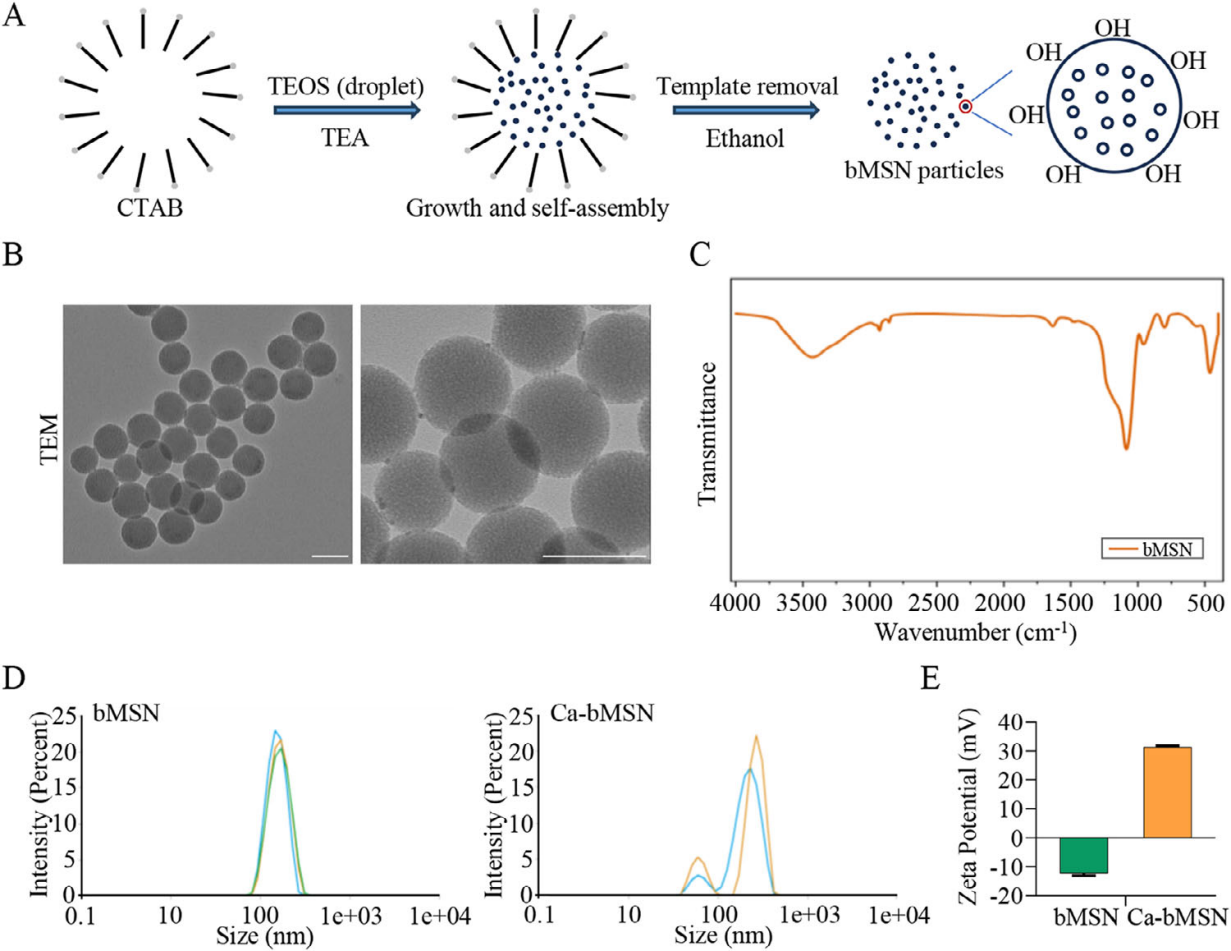
Preparation and physicochemical characterization of bMSN and Ca‐bMSN. A) Schematic of bMSN synthesis processes. B) TEM images of bMSN. Scale bar = 200 nm. C) FTIR spectra of bMSN. D) Size of bMSN and Ca‐bMSN. E) Potential of bMSN and Ca‐bMSN.

Next, we attempted to evaluate the protective effect of Catalpol on OA in vivo. Injecting drugs into the joint cavity is a highly effective clinical treatment strategy with fewer side effects. However, the efficacy of drugs may be reduced due to their rapid clearance from the joint cavity. Thus, to prolong the action time of Catalpol in vivo, the bMSNs—large surface area, excellent biocompatibility, and high drug loading capacity—were used to deliver Catalpol. **Figure** **3**A schematically illustrates the bMSN synthesis process. Transmission electron microscopy (TEM) imaging revealed well‐distributed, uniformly sized, and spherical bMSNs (Figure 3B). Fourier transform infrared (FTIR) spectroscopy confirmed the presence of the characteristic Si─O─Si bond in bMSNs (Figure 3C). The bMSNs exhibited a particle size of ≈200 nm and a zeta potential of −12.42 mV (Figure 3D). Notably, the lower zeta potential of Ca‐bMSN (31.3 mV) compared to bMSN (Figure 3E) indicated successful loading of Catalpol onto the bMSNs. High‐performance liquid chromatography (HPLC) analysis determined a drug loading efficiency (LE) of 35% (Figure S3, Supporting Information).

### 2.4 Assessment of the Cellular Uptake and Chondroprotective Effect of Ca‐bMSN In Vitro

**Figure 4:**
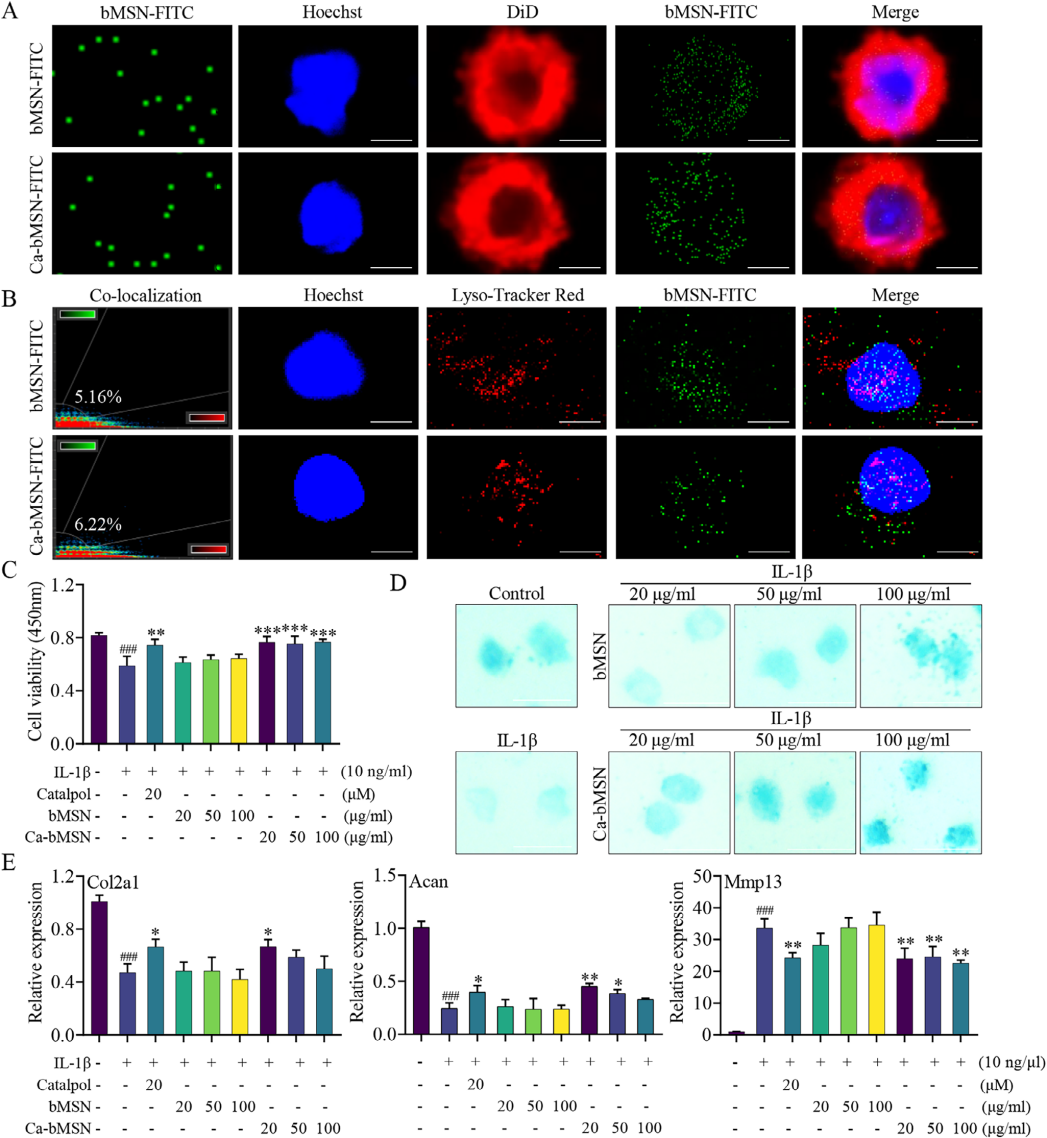
Ca‐bMSN promotes anabolism in primary chondrocytes and inhibits catabolism. A) Ca‐bMSN and bMSN labeled with FITC observed by confocal laser microscope (left). Uptake of bMSN and Ca‐bMSN by IL‐1β induced primary chondrocytes. Nuclei, blue; Cell membrane, red (right). Scale bar = 5 µm. B) Fluorescent visualization (right) co‐localization between bMSN or Ca‐bMSN and lysosome in IL‐1β induced primary chondrocytes. Ca‐bMSN or bMSN, green; Nuclei, blue; Lysosome, red. The quantification result is shown at left. Scale bar = 5 µm. C) The effect of bMSN or Ca‐bMSN on primary chondrocytes viability was detected by CCK‐8 (*n* = 3, *p < 0.05, **p < 0.01, ### or ***p < 0.001 when compared with control group and IL‐1β group, respectively). D) Effects of bMSN and Ca‐bMSN on cell morphology and the synthesis of proteoglycan in chondrocytes. Scale bar = 50 µm. E) The expression level of *Col2a1*, *Acan*, and *Mmp13* in the primary chondrocytes, which were stimulated with IL‐1β combined with Catalpol, bMSN, or Ca‐bMSN treatment for 24 h (*n* = 3, *p < 0.05, **p < 0.01, ### or ***p < 0.001 when compared with control group and IL‐1β group, respectively). Data are shown as mean ± SEM. *P*‐values were obtained by one‐way ANOVA with multiple comparisons.

Before in vivo experiments, we incubated chondrocytes with fluorescein isothiocyanate (FITC)‐modified bMSN and Ca‐bMSN. Laser confocal microscopy revealed that both bMSN and Ca‐bMSN were efficiently taken up by mouse chondrocytes that were treated with IL‐1β within 4 h, as well as in mouse and human chondrocytes (**Figures** **4**A and S4A, Supporting Information). This suggests these nanoparticles could be taken up by chondrocytes. Macromolecules and nanoparticles typically enter cells via endocytosis, followed by trafficking to lysosomes for degradation. Lysosome co‐localization experiments demonstrated that only a minimal amount of internalized bMSN and Ca‐bMSN (5.16%, 6.22%) signals co‐localized with lysosomes in the mouse chondrocytes that were treated with IL‐1β (Figures 4B and S4B, Supporting Information). This suggests that bMSN and Ca‐bMSN could bypass the traditional endocytic pathway and evade degradation within lysosomes. Furthermore, bMSN at concentrations of 20, 50, and 100 µg mL^−1^ exhibited no toxic effects on primary chondrocytes and Ca‐bMSN at 20 and 50 µg mL^−1^ significantly promoted chondrocyte proliferation (Figure 4C). In addition, both bMSN and Ca‐bMSN treatments maintained normal cell morphology and high ECM synthesis ability, suggesting no obvious toxic effects on the cells (Figure 4D). We further investigated the expression levels of *Col2a1, Acan*, and *Mmp13* in primary chondrocytes treated with IL‐1β, IL‐1β + Catalpol, and IL‐1β + Ca‐bMSN (using a concentration of Ca‐bMSN equivalent to 20 × 10^−6^ m Catalpol). The results demonstrated that 20 µg mL^−1^ Ca‐bMSN displayed effects comparable to 20 × 10^−6^ m Catalpol, promoting *Col2a1* and *Acan* expression while inhibiting *Mmp13* expression (Figure 4E).

### 2.5 The In Vivo Chondroprotective Efficacy of Catalpol and Ca‐bMSN

Degradable MSNs are generally considered preferable to nondegradable ones for medical applications due to their lower bioaccumulation and higher biocompatibility. To investigate whether Ca‐bMSN could deliver drugs to cartilage and undergo biodegradation, we monitored the signal from bMSN and Ca‐bMSN in rat knee joints after successful injection (Figure S5A, Supporting Information). MSNs have been reported to be as CT and optical imaging agents, thus the degradation of bMSN and Ca‐bMSN in rat knee joints was monitored at day 0, 1, 3, 5, and 7 by micro‐CT. The results showed that both bMSN and Ca‐bMSN resided in the joint for ≈7 d before degrading (Figure S5B, Supporting Information). TEM images revealed a noticeable alteration in the morphology of bMSNs, with a rapid decrease in nanoparticle diameter and minimal residual material detectable by day 7. In contrast, the morphology of MSNs remained largely unchanged from day 0 to day 7 (Figure S5C, Supporting Information). Furthermore, HPLC analysis was used to determine the retention of Catalpol in OA articular fluid at predetermined time points. This analysis revealed that the Ca‐bMSN group exhibited significantly higher Catalpol retention compared to the Catalpol‐alone group even at 24 h. This suggests that Ca‐bMSN effectively achieves sustained release of Catalpol within the articular fluid of OA rats (Figure S5D, Supporting Information).

**Figure 5:**
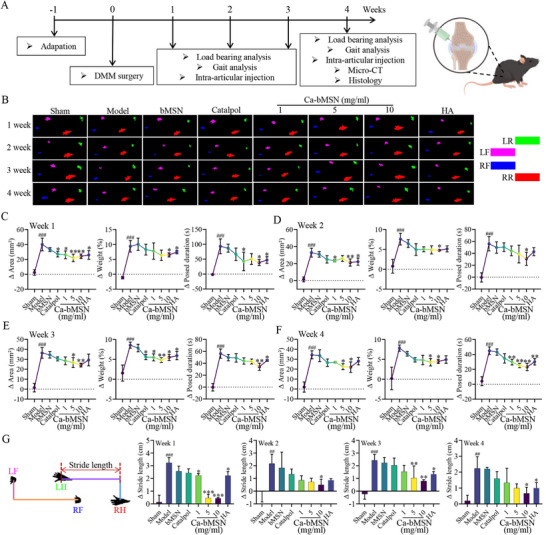
Behavioral effects of Catalpol, bMSN, and Ca‐bMSN treatment on OA rat. A) The schematic diagram depicts the process of creating a rat model of OA and the experimental design used to assess the protective effects of Catalpol, bMSN, and Ca‐bMSN. HA served as positive control. B) Gait analysis images of Sham, Model, bMSN, Catalpol, Ca‐bMSN, and HA groups. LF, left front paw; RF, right front paw; LR, left rear paw; RR, right rear paw. Δ Area, Δ Weight, and Δ Posed duration of sham, model Catalpol, bMSN, Ca‐bMSN, and HA groups at C) week 1, D) week 2, E) week 3, and F) week 4 (*n* = 5, *p < 0.05, **p < 0.01, ### or ***p < 0.001 when compared with sham group and model group, respectively). G) Δ Stride length of sham, model Catalpol, bMSN, Ca‐bMSN, and HA groups (*n* = 5) is shown during a gait cycle. # versus Sham, * versus Model, *p < 0.05, ## or **p < 0.01, ### or ***p < 0.001 (*n* = 5, *p < 0.05, ## or **p < 0.01, ### or ***p < 0.001 when compared with sham group and model group, respectively). Data are shown as mean ± SEM. *P*‐values were obtained by one‐way ANOVA with multiple comparisons.

Behavioral tests were undertaken to assess the cartilage protection efficacy of Catalpol and Ca‐bMSN at different concentrations (1, 5, 10 mg mL^−1^) on OA model rats (**Figure** **5**A). Hyaluronic acid (HA), a common clinical treatment strategy for OA, was served as positive control. Gait analysis revealed a significant increase in paw area for rats treated with Catalpol alone, all Ca‐bMSN concentrations (1, 5, 10 mg mL^−1^) and HA compared to the OA model group and the free bMSN group (Figure 5B). Treatment with Catalpol, various Ca‐bMSN concentrations, and HA significantly reduced the differences in bearing area (Δarea), bearing weight (Δweight), and swinging time (Δposture duration) between the left and right hind limbs of OA model rats compared to the model group, free bMSN did not demonstrably affect Δarea, Δweight, or Δposture duration in OA model rats (Figure 5C–F). Furthermore, we compared the walking length discrepancies between the left and right hind limbs of OA rats. The findings indicated that as the treatment progressed from week 1 to week 4, the walking length difference tended to disappear, particularly in the high‐dose Ca‐bMSN group, which exhibited a superior therapeutic effect compared to Catalpol, low‐dose Ca‐bMSN, medium‐dose Ca‐bMSN and HA groups, bMSN alone did not have any significant impact on the walking length of OA model rats (Figure 5G). These results collectively demonstrate that Ca‐bMSN offers significantly greater protective effects against OA symptoms like pain and limited joint mobility compared to bMSN alone, Catalpol alone, or HA.

**Figure 6:**
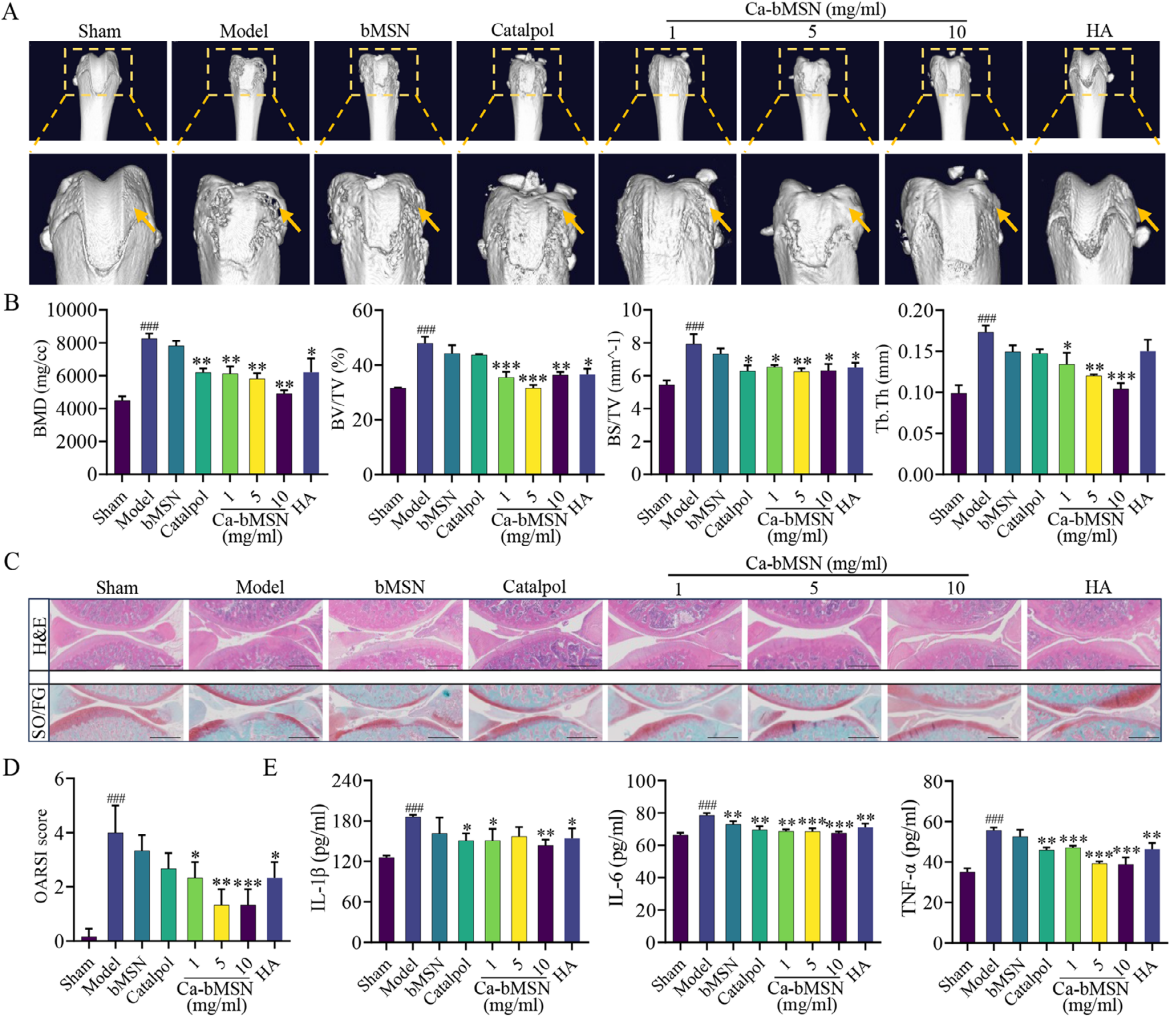
In vivo effect of Catalpol, bMSN, and Ca‐bMSN treatment on OA rat. A) 3D images of the knee joints by micro‐CT at 4 weeks of treatment with Catalpol, bMSN, Ca‐bMSN, and HA groups. Arrows indicate the lesion points of joint. B) Quantitative analysis of BMD, BV/TV, BS/TV, and Tb.Th (*n* = 5, *p < 0.05, **p < 0.01, ### or ***p < 0.001 when compared with sham group and model group, respectively). C) H&E staining and safranin‐O‐fast green staining (SO/FG) of knee joints treated with Catalpol, bMSN, Ca‐bMSN, and HA at 4 weeks. Scale bar = 500 µm. D) The severity of OA in the sham, model Catalpol, bMSN, Ca‐bMSN, and HA groups was analyzed by grading histological sections using the Osteoarthritis Research Society International (OARSI) score system (*n* = 5, *p < 0.05, ## or **p < 0.01, ### or ***p < 0.001 when compared with sham group and model group, respectively). E) The concentration of IL‐1β, IL‐6, and TNFα in joint fluid of sham, model, Catalpol, bMSN, Ca‐bMSN, and HA groups (*n* = 5, *p < 0.05, ## or **p < 0.01, ### or ***p < 0.001 when compared with sham group and model group, respectively). Data are shown as mean ± SEM. *P*‐values were obtained by one‐way ANOVA with multiple comparisons.

The subchondral bone undergoes structural changes during OA development. Administration of Catalpol and various concentrations of Ca‐bMSN effectively inhibited abnormal bone remodeling within the subchondral bone (**Figure** **6**A). Compared to the OA model group, treatment groups exhibited significantly lower values for bone mineral density (BMD), bone volume fraction (BV/TV), bone surface fraction (BS/TV), and trabecular thickness (Tb.Th) (Figure 6B). Histological analysis using hematoxylin and eosin (H&E) and safranin‐O‐fast green staining revealed a thinner cartilage layer with a coarse surface in the OA model group. In contrast, the Catalpol and Ca‐bMSN groups displayed a smooth cartilage surface with well‐organized chondrocytes, particularly in the high‐dose Ca‐bMSN groups (5 and 10 mg mL^−1^) (Figure 6C). Consequently, Ca‐bMSN at all concentrations (1, 5, and 10 mg mL^−1^) had lower OARSI scores compared to the free Catalpol, bMSN, HA, and OA model groups (Figure 6D). Furthermore, Catalpol, Ca‐bMSN, and HA treatment reversed the increase in inflammatory mediators (IL‐1β, IL‐6, and TNF‐α) observed in the joint fluid of OA rats compared to other groups (Figure 6E). Notably, bMSN alone did not cause an inflammatory reaction in joint fluid. Immunohistochemistry (IHC) and immunofluorescence analyses revealed that Catalpol and Ca‐bMSN treatment decreased the expression of MMP9, MMP13, and ADAMTS4, while increasing the expression of COL2A1 and ACAN in OA rats compared to other groups (Figure S6A,B, Supporting Information).

To further determine whether the administration of Catalpol and different concentrations of Ca‐bMSN (1, 5, 10 mg mL^−1^) have the same cartilage protective effect in the OA mouse model as in the OA rat model, gait analysis, micro‐CT, and histological analyses were performed. As shown in Figure S7A–E (Supporting Information), Catalpol, various Ca‐bMSN concentrations, and HA significantly reduced the differences in bearing area (Δarea), bearing weight (Δweight), and swinging time (Δposture duration) between the left and right hind limbs of OA mouse model compared to the model group. However, bMSN alone did not have a significant impact on gait parameters. Micro‐CT analysis revealed an increase in knee joint bone hyperplasia and osteophyte formation in the OA mouse model group, along with elevated subchondral bone parameters such as BMD, BV/TV, BS/TV, and Tb.Th, compared to the sham group. Treatment with Catalpol and varying concentrations of Ca‐bMSN effectively inhibited hyperplasia, osteophyte formation, and subchondral bone abnormal remodeling, as illustrated in Figure S7F,G (Supporting Information). Furthermore, Catalpol, Ca‐bMSN, and HA administration mitigated polysaccharide loss and cartilage surface damage, as evidenced by Safranin O‐Fast Green (SO/FG) staining, shown in Figure S7H (Supporting Information). These results suggested that Catalpol and Ca‐bMSN could significantly ameliorate the degree of joint cartilage degradation and improve joint function in the OA mouse model, as in the OA rat model.

To evaluate the potential toxicity of bMSN and Ca‐bMSN, histopathological analysis was conducted on liver, spleen, kidney, lung, and heart tissues obtained from rats subjected to intra‐articular injections of bMSN and high‐concentration Ca‐bMSN. No histopathological abnormalities were detected in these organs (Figure S8, Supporting Information). In addition, the hemolysis percentage of bMSN and Ca‐bMSN was determined to be less than 5% (Figure S9A,B, Supporting Information). The biochemical indicators, including ALT, AST, ALP, and CREA, were performed to assess the potential toxicity towards the major organs of bMSN and Ca‐bMSN. As shown in Figure S9C,D (Supporting Information), bMSN and Ca‐bMSN have no potential liver and kidney toxicity in rat and mouse. Taken together, these data demonstrate that Ca‐bMSN has the therapeutic potential to improve OA progression in knee OA animal model without obvious side effects.

### 2.6 Identification of Potential Molecular Mechanisms Regulated by Catalpol

**Figure 7:**
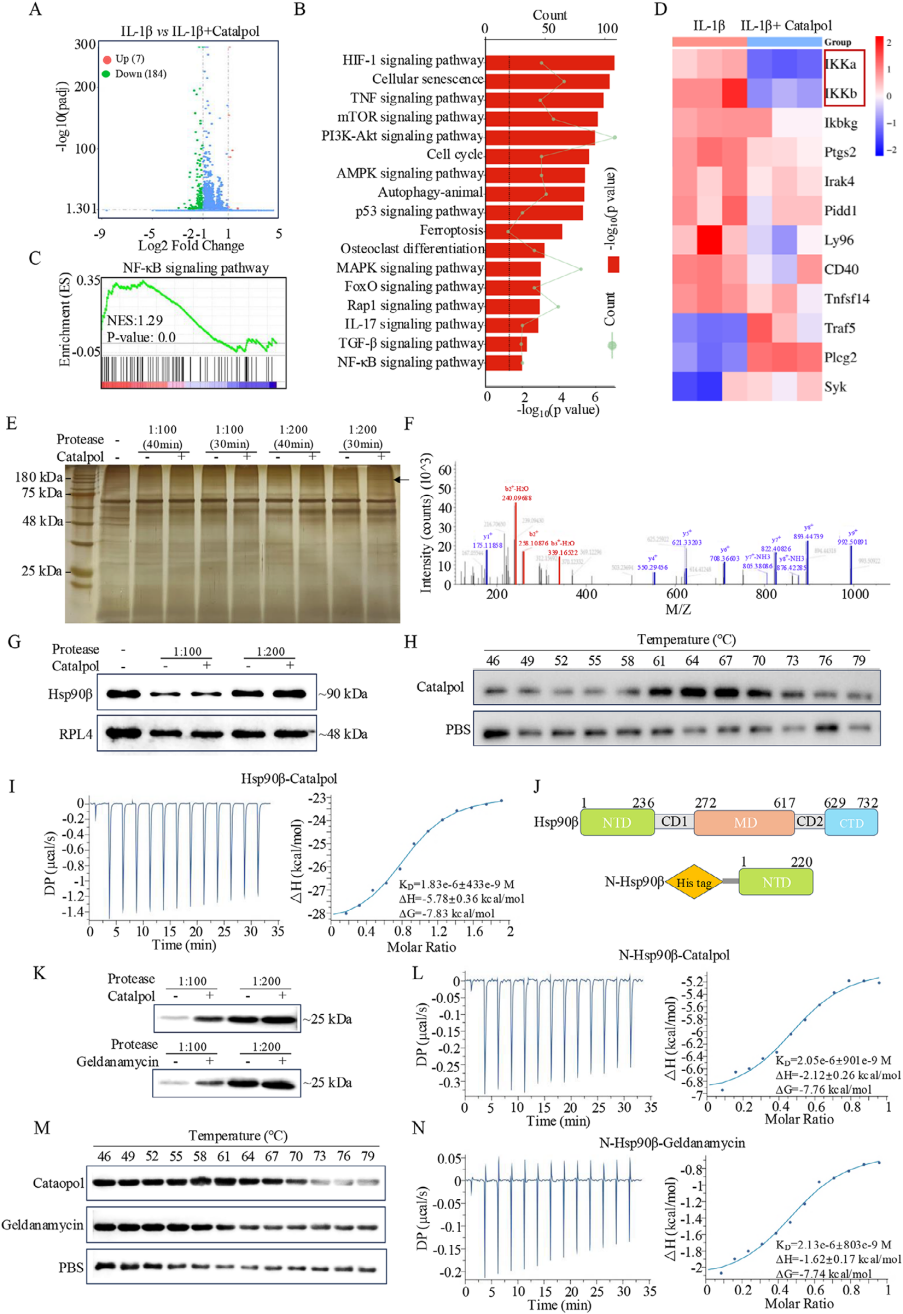
Hsp90β was identified as the target of Catalpol. A) Volcano plot representation of the different expression genes. Green dots: downregulated genes, red dots: upregulated genes, blue dots: none. B) KEGG pathway enrichment analysis. C) GSEA enrichment analysis indicated that Catalpol regulates NF‐κB signaling pathway. D) Heat map of gene expression associated with NF‐κB signaling pathway. E) The DARTS samples were subjected to silver staining, revealing that Catalpol provided protection to the band with a molecular weight of ≈180 kDa. F) Mass spectrogram of Hsp90β protein. G) Total protein from primary chondrocytes were treated with varying doses of protease with or without Catalpol, followed by the detection of Hsp90β levels through Western blot analysis. H) Mouse primary chondrocytes lysate was incubated with PBS or Catalpol for 30 min at 25 °C, and then denatured under various temperatures. The protein level of Hsp90β was assayed by Western blot. I) The affinity between Hsp90β and Catalpol was measured by ITC. J) Domain structure of HSP90 (732 aa) and N‐terminal domain of HSP90 (N‐Hsp90, 1–220 aa). K) N‐terminal of Hsp90β (1–220 aa) was treated with varying doses of protease with or without Catalpol or Geldanamycin, followed by the detection of Hsp90β levels through Western blot analysis. L) The affinity between the N‐terminal of Hsp90β (1–220 aa) was measured by ITC. M) N‐terminal of Hsp90β (1–220 aa) was incubated with PBS, Catalpol, or Geldanamycin for 30 min at 25 °C, and then denatured under various temperatures. The protein level of N‐terminal of Hsp90β (1–220 aa) was assayed by Western blot. N) The affinity between the N‐terminal of Hsp90β (1–220 aa) and Geldanamycin was measured by ITC.

To illuminate the underlying therapeutic mechanism of Catalpol, RNA‐seq was performed. The differential gene expression profile was visualized using a volcano plot (**Figure** **7**A), revealing seven significantly upregulated genes and 184 significantly downregulated genes in the IL‐1β versus IL‐1β + Catalpol group. KEGG pathways enrichment observed 17 pathways were significantly enriched, mainly involved in PI3K‐Akt, cellular senescence, MAPK, NF‐κB, etc. It is noteworthy that PI3K/Akt and MAPK pathways were involved in the activation of NF‐κB (Figure 7B). Furthermore, the gene set enrichment analysis (GSEA) further proved that the NF‐κB signaling pathway may be regulated by Catalpol (Figure 7C). The heat map in Figure 7D demonstrated noteworthy differences genes in the NF‐κB signaling pathway in the IL‐1β versus IL‐1β + Catalpol group. Therefore, the NF‐κB pathway was more critical. Moreover, transcriptome analysis confirmed that Catalpol regulates the genes expression level involved in cell apoptosis, oxidative stress, and ECM synthesis and degradation (Figure S10, Supporting Information). While NF‐κB signaling pathway could regulate cell apoptosis, oxidative stress, and ECM degradation. These results indicated that Catalpol exerts anti‐OA effects that may be mediated by the modulation of the NF‐κB signaling pathway.

### 2.7 HSP90β Is a Novel Target of Catalpol

To identify the target of Catalpol in chondrocytes, we performed a drug affinity responsive target stability (DARTS) assay on primary chondrocyte protein extracts, followed by analysis using SDS‐PAGE. A comparison of Catalpol and control lysate lanes on the gel revealed a band with a molecular weight of ≈180 kDa that exhibited different intensities (Figure 7E). These bands were excised for unbiased, high‐throughput protein identification by mass spectrometry (Figure 7F). Mass spectrometry identified a total of 202 proteins. Hsp90β displayed the highest significance of difference between Catalpol and control groups with high confidence, suggesting it as a potential target candidate. To confirm Hsp90β as Catalpol's target, we conducted DARTS and Western blot assays. Chondrocyte total protein was digested with varying protease concentrations, with or without Catalpol incubation. Our results suggest that Catalpol protects Hsp90β from enzymatic degradation (Figure 7G). A cellular thermal shift assay (CETSA) was performed to further validate the interaction between Hsp90β and Catalpol. Compared to the PBS group, Catalpol significantly increased Hsp90β accumulation at temperatures of 61, 64, 67, 70, and 73 °C, suggesting that Catalpol enhances Hsp90β stability (Figure 7H). Isothermal titration calorimetry (ITC) confirmed direct binding between Catalpol and Hsp90β with a dissociation constant (*K*_D_) of 1.83 × 10^−6^ ± 433 × 10^−9^ m (Figure 7I). Since Hsp90β inhibitors typically target the ATP‐binding site of the N‐terminal domain, the N‐terminal domain of Hsp90β (N‐Hsp90β, amino acids 1–220) was produced and used to assess its binding capacity with Catalpol (Figure 7J). Geldanamycin, a known Hsp90 inhibitor targeting the ATP‐binding domain, was used as a positive control. DARTS and CETSA assays demonstrated the protective effects of both Catalpol and Geldanamycin on Hsp90β against enzymatic digestion and high‐temperature‐induced degradation or denaturation (Figure 7K,M). In addition, ITC analysis determined the binding affinities between N‐Hsp90β and Catalpol or Geldanamycin to be ≈2.05 × 10^−6^ and 2.13 × 10^−6^ m, respectively (Figure 7L,N). Taken together, these findings demonstrate that Catalpol could directly bind to Hsp90β, particularly through its interaction with the N‐terminal domain.

### 2.8 Molecular Docking, MM‐GBSA Calculation, and Molecular Simulation

**Figure 8:**
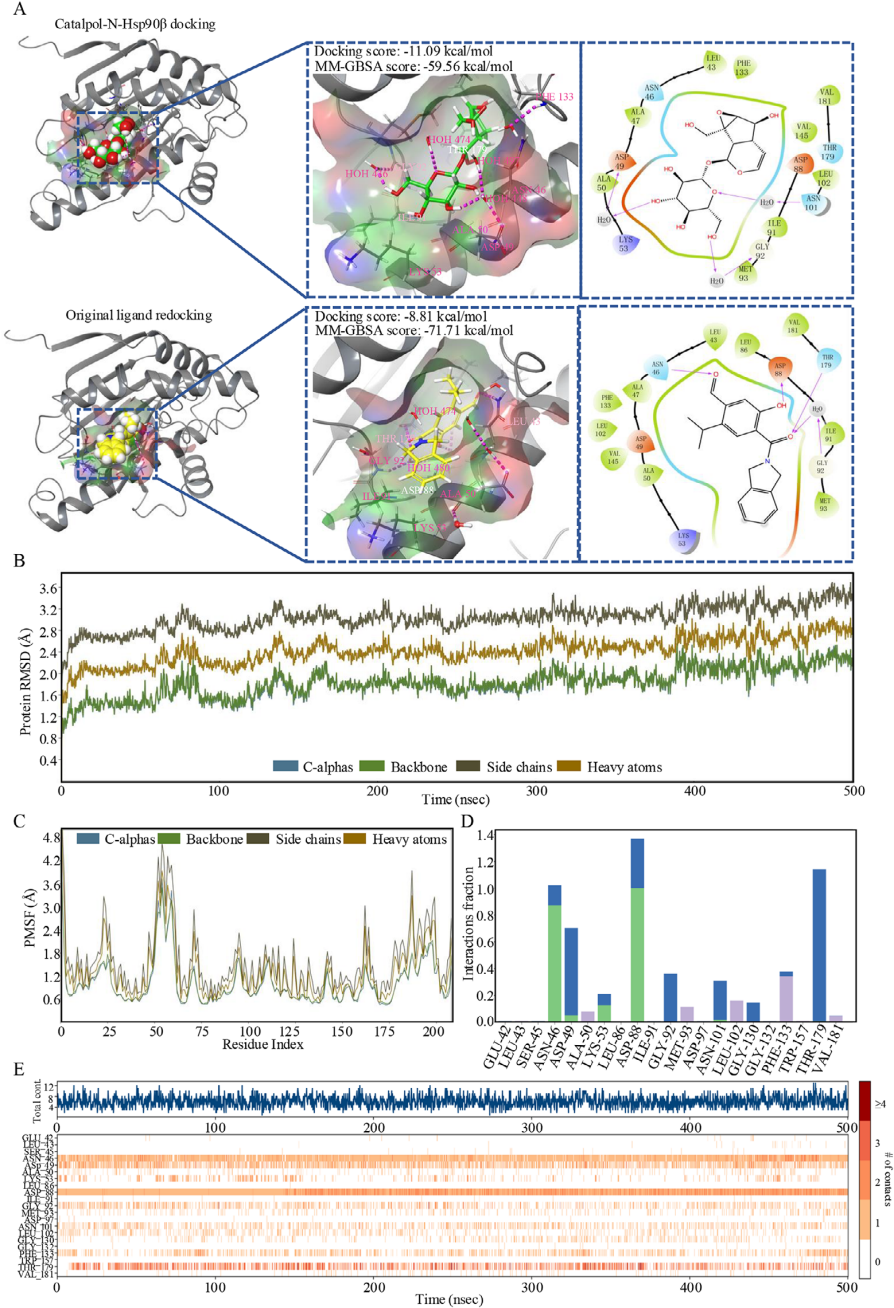
Hsp90β molecular simulations. A) Binding model of Catalpol into the N‐terminal ATP‐binding site of Hsp90β and original ligand redocking. A diagram illustrating the precise atomic interactions between the ligand and Hsp90β residues over a 500 ns molecular dynamic simulation. Only the interactions that occurred for more than 30.0% of the simulation time within the chosen trajectory are depicted. B) RMSD plots of protein C‐alpha, backbone, side chain, heavy atoms throughout the 500 ns MD simulations. C) RMSF plots of Hsp90β‐Catalpol during the MD simulations. D) The statistical graph illustrates protein–ligand interactions between Hsp90β and Catalpol. The value indicates the percentage of time the specific interaction is maintained during the simulation. Values above 1.0 signify that the relative residue establishes multiple contacts with Catalpol. E) The timeline representation of the interactions and contacts. The upper panel illustrates the cumulative count of distinct interactions established by Hsp90β with the ligand throughout the trajectory. Conversely, the lower panel delineates the specific residues engaged in interactions with Catalpol in each frame of the trajectory. Notably, these residues exhibit multiple specific contacts with Catalpol, as indicated by the darker shade of orange on the color scale provided alongside the plot.

A docking simulation was performed to gain a deeper understanding of the interaction between Catalpol and the N‐terminal domain of Hsp90β (N‐Hsp90β). This simulation aimed to predict Catalpol's binding affinity and identify the critical amino acids forming the binding site. As shown in **Figure** **8**A, the N‐Hsp90β‐Catalpol complex displayed water‐mediated hydrogen bonds between residues GLY92, ASN101, ASP49 of N‐Hsp90β and the hydroxyl groups of Catalpol's sugar moiety. Notably, Catalpol exhibited a stronger binding affinity (−11.09 kcal mol^−1^) toward N‐Hsp90β compared to the original ligand (−8.81 kcal mol^−1^), likely due to this water‐mediated hydrogen bond network. Prime MM‐GBSA calculations were used to accurately predict the binding energy of the docked pose. The binding energy (Gbind) of Catalpol was −59.56 kcal mol^−1^, which was higher than the original ligand (−71.71 kcal mol^−1^). To further validate the binding free energy prediction, a MD simulation was conducted. The root‐mean‐square deviation (RMSD) of the N‐Hsp90β‐Catalpol complex remained within a stable range of 1–3 Å throughout the simulation (Figure 8B), suggesting rapid stabilization around 1.5 Å within 5 ns. This indicated that Catalpol has a strong stabilizing effect on N‐Hsp90β. The RMSD analysis supports the accuracy of the docking results and suggests that the complex achieved a stable conformation during the simulation. Furthermore, root mean square fluctuation (RMSF) analysis revealed significantly low fluctuations for residues (ASP88, GLY92, THR179) located around the binding pocket (Figure 8C). This suggests these residues are crucial for intermolecular interactions, thereby stabilizing the protein conformation. Intermolecular interaction analysis identified four types of contacts between Catalpol and N‐Hsp90β: hydrogen bonds, hydrophobic interactions, ionic bonds, and water bridges. The hydroxyl group at the C6 position of Catalpol's sugar moiety acted as a hydrogen bond donor with ASN46 (Figure 8D). In addition, water bridges contributed to contacts between ASP49 and the C6 hydroxyl group, which represented a vital functional group for Catalpol's inhibitory activity. The hydroxyl group on the side chain of the iridoid scaffold also interacted with N‐Hsp90β in two ways: as a hydrogen bond donor with ASP88 and through a water bridge with THR179. Interestingly, the epoxy group in the iridoid structure also interacted with the crucial amino acid ASP88 via a water bridge. Based on the interaction statistics obtained during the 500 ns simulation, it is highly conceivable that ASN46, ASP88, and THR179 play significant roles in forming a stable binding conformation (Figure 8E). In conclusion, the findings from the MD simulation are consistent with the ITC experiments, suggesting that Catalpol has the potential to act as an Hsp90β inhibitor.

### 2.9 Catalpol Regulates Chondrocytes Metabolism and Oxidative Stress via Inhibition of Hsp90β/NF‐κB Signaling Pathway

**Figure 9:**
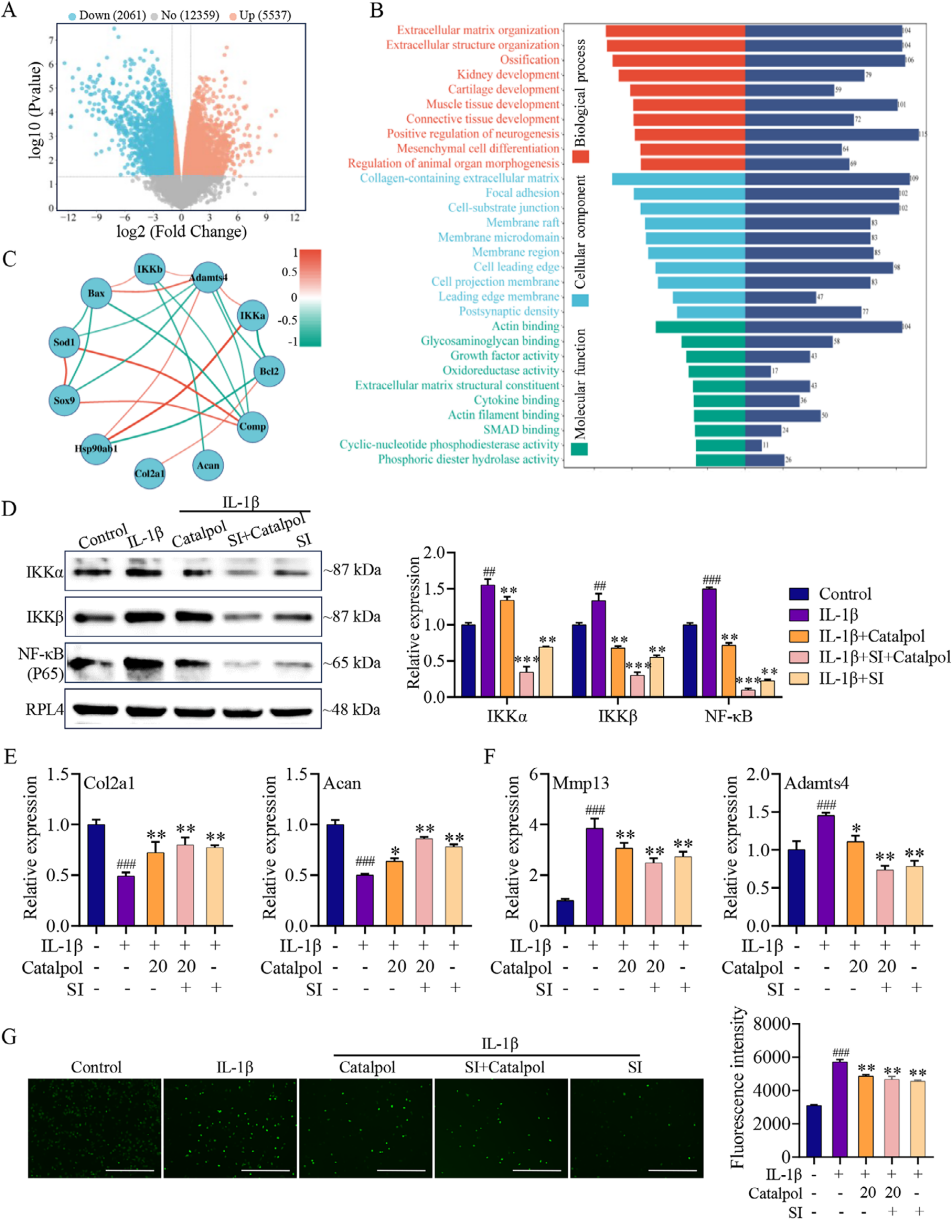
Hsp90β is required for Catalpol regulation of chondrocyte metabolism and ROS production induced by IL‐1β through the NF‐κB signaling pathway. A) Volcano plot representation of the different expression genes in patents chondrocytes and health chondrocytes. B) Differential expression genes GO enrichment. C) Correlation analysis to analyze the correlation between target gene *Hsp90ab1* and chondrocytes apoptosis (*Bax, Bcl2*), oxidative stress (*Sod1*), ECM metabolism (*Sox9, Col2a1, Acan, Comp*), and NF‐κB signaling pathway (*IKKα, IKKβ*) marker genes. Red: positive correlation; Blue: negative correlation. D) The changes of NF‐κB signaling pathway between different groups using Western blot assay. The quantification result is shown at right (*n* = 3, ## or **p < 0.01, ### or ***p < 0.001 when compared with control group and IL‐1β group, respectively). E,F) qRT‐PCR analysis of the expression level of anabolism marker genes (*Col2a1, Acan*) and catabolism marker genes (*Mmp13, Adamts4*) in chondrocytes with or without IL‐1β, Catalpol, or *Hsp90ab1*‐siRNA (SI) (*n* = 3, *p < 0.05, **p < 0.01, ###*p* < 0.001 when compared with control group and IL‐1β group, respectively). G) ROS production in chondrocytes under the treatment with IL‐1β or IL‐1β combined with Catalpol, SI+ Catalpol, and SI was determined by DCFH‐DA. Fluorescence microscopy was used to capture images (left). ROS production was determined by the DCF fluorescence intensity was determined by microplate reader (right). Scale bar = 400 µm (*n* = 3, **p < 0.01, ###*p* < 0.001 when compared with control group and IL‐1β group, respectively). Data are shown as mean ± SEM. *P*‐values were obtained by one‐way ANOVA with multiple comparisons.

To explore the potential correlation between Hsp90β and chondrocyte apoptosis, oxidative damage, anabolism/catabolism and NF‐κB signaling pathway in knee OA patients, the publicly accessible transcriptomic data of aged male OA chondrocytes and healthy male chondrocytes sourced from the Gene Expression Omnibus (GEO) database (GSE 246425) was subjected to reanalysis. The volcano plots revealed that 5537 genes were significantly upregulated and 2061 genes were downregulated in old OA male chondrocytes versus OA health male chondrocytes group, and *Hsp90ab1* is highly expressed in OA patients (**Figure** **9**A and Figure S11A, Supporting Information). Gene ontology (GO) annotations described the biological roles of differential genes sorted as three categories including biological process, cellular component, and molecular function, mainly including extracellular matrix organization, extracellular structure organization, collagen containing extracellular, actin binding process, and so on (Figure 9B). Gene correlation analysis revealed significant positive correlations between *Hsp90ab1* and *IKKα*, as well as *Adamts4*, and a negative correlation with *Bcl2*. In addition, *IKKα* exhibited positive correlations with *Adamts4*, *IKKβ*, and *Bax*, while *Bax* displayed negative correlations with *Sod1, Sox9*, and *Comp* (Figure 9C). The findings suggest a positive correlation between *Hsp90ab1* and marker genes associated with ECM catabolism, apoptosis, oxidative damage, and the NF‐κB signaling pathway, as well as a negative correlation with marker genes related to ECM anabolism, anti‐apoptosis, and anti‐oxidation.

During OA development, IL‐1β stimulation promotes oxidative stress, degradation of ECM, and through the activation of the NF‐κB pathway. To investigate whether Catalpol protects chondrocytes by targeting Hsp90β, leading to the inhibition NF‐κB signaling pathway, protein–protein interactions among the Hsp90β, IKKβ, IKKα, NF‐κB (p65), MMP13, ADAMTS4, COL2A1, and ACAN were first predicted with the STRING tool and revealed that Hsp90β served as a hub protein and had the strongest interaction with IKKβ, IKKα, and NF‐κB (p65), and then regulated other genes associated with ECM metabolism (Figure S11B, Supporting Information). Subsequently, we treated an OA chondrocyte model with Catalpol alone, Catalpol combined with siRNA targeting Hsp90β (siRNA‐*Hsp90ab1*, SI), or SI alone. *Hsp90ab1* gene silence efficacy by siRNA is shown in Figure S11C (Supporting Information). Western blot analysis was then performed to assess the total protein expression of IKKα, IKKβ, and P65. As shown in Figure 9D, silencing the expression of *Hsp90ab1* gene in the primary chondrocytes was accompanied by the decreased expression of IKKα, IKKβ, and NF‐κB (P65) protein. Meanwhile, Catalpol treatment, either alone or combined with SI, significantly suppressed the expression levels of IKKα, IKKβ, and P65 compared to untreated controls. To validate the working mechanism of Catalpol in vivo, 6 nmol siRNA‐*Hsp90ab1*, processed with ago‐modification, were injected into OA rat joint cavity weekly. Hsp90ab1 gene silence efficacy by siRNA is shown in Figure S11D (Supporting Information). The expression level of IKKα, IKKβ, and NF‐κB (P65) was detected by Western blot. As shown in Figure S12 (Supporting Information), the same as in vitro results above were also obtained in in vivo experiments. In addition, Catalpol treatment alone or combined with SI significantly upregulated the expression of anabolic marker genes (*Col2a1* and *Acan*) and downregulated the expression of catabolic marker genes (*Mmp13* and *Adamts4*) (Figure 9E,F). Meanwhile, ROS levels were significantly reduced in chondrocytes treated with Catalpol, Catalpol combined with SI, or SI alone (Figure 9G). Notably, knockdown of *Hsp90ab1* in primary chondrocytes demonstrates a similar biological effect as Catalpol treatment on cell metabolic activity and ROS level, and the combined administration of Catalpol and SI does not result in a more pronounced regulatory effect than SI alone, potentially due to the absence of Hsp90β protein. These findings demonstrate that Catalpol exhibits protection effects on chondrocytes specifically by targeting Hsp90β, leading to the inhibition of the NF‐κB signaling pathway.

## 3 Discussion

OA is the predominant chronic degenerative condition affecting articular cartilage, characterized by chronic pain, functional limitations, instability, and deformity, resulting in a significant decline in quality of life. Globally, OA is a leading cause of disability among older individuals.^[^ ^13^ ^]^ Due to its high prevalence and associated disability, OA imposes a significant burden on both individuals and society. Restore the normal architecture and function of a damaged joint by the chondrogenic differentiation of stem cells and the synthesis of cartilage matrix by chondrocytes are the effective strategies for OA treatment. Currently, several effective protein drugs have been developed for this strategy. LNA043, a recombinant version of the human angiopoietin‐like 3 (ANGPTL3) protein, was reported to facilitate the chondrogenic differentiation of MSCs and enhanced the anabolic activities of cartilage, that is currently in a phase 2b trial (NCT04864392) in patients with knee OA.^[^ ^14^ ^]^ Sprifermin (recombinant human FGF18, rhFGF18) can not only promote the proliferation of chondrocytes and the synthesis of ECM, but also inhibit the activity of proteolytic enzymes and remarkedly reduce the degeneration of cartilage, which is currently in phase III clinical trial.^[^ ^15^ ^]^ TCM has been shown to be effective and safe for treating OA. There is a growing interest in exploring compounds derived from natural plants or traditional medicinal practices as potential treatments for OA due to their demonstrated clinical efficacy and minimal side effect profile. However, the development of traditional Chinese medicine monomer still lags behind that of protein drugs.

Catalpol emerged as a candidate with the ability to upregulate the expression of anabolic marker genes (*Col2a1*, *Acan*, *Comp*) and downregulate the expression of catabolic marker genes (*Mmp9*, *Mmp13*, *Adamts4*) in human and mouse chondrocytes. In vivo administration of Catalpol demonstrated efficacy in limiting OA development, alleviating OA‐associated pain sensitivity, and partially reversing cartilage degradation in OA rat models. To elucidate the mechanisms underlying Catalpol's protective effect, we used a comprehensive suite of techniques including DARTS assays, proteomics, CETSA, ITC, molecular dynamics simulations, and RNA‐seq experiments. These investigations revealed Catalpol treatment correlated with NF‐κB signaling pathway and Hsp90β as the direct target of Catalpol, with a binding affinity of 1.83 × 10^−6^ ± 433 × 10^−9^ m.

Hsp90β, a member of the large heat shock protein (HSP) family, functions as a molecular chaperone. It plays a critical role in protein folding, stabilization, and the refolding of denatured proteins.^[^ ^16^, ^17^ ^]^ Previous studies have demonstrated that Celastrol, a triterpenoid compound, inhibits the activity of Hsp90β and suppresses the production of MMPs, inducible nitric oxide synthase (iNOS), and cyclooxygenase‐2 (COX‐2) in chondrocytes isolated from osteoarthritic patients.^[^ ^18^ ^]^ Furthermore, Hsp90β has been shown to reduce the levels of NO production and chondrocyte death induced by IL‐1β or TNF‐α.^[^ ^19^ ^]^ The present study confirmed that knockdown of *Hsp90ab1* expression could significantly upregulate chondrocyte anabolic activity. In addition, our findings demonstrated that the chondroprotective effect of Catalpol was dependent on *Hsp90ab1*. In conjunction with these observations and previous reports, we propose that *Hsp90ab1* represents a promising drug target for the treatment of OA. Hsp90β exhibited a high degree of evolutionary conservation across species. The amino acid sequences of Hsp90β were 100% identical between mice and rats, and only three amino acid residues differ between humans and these rodents (Figure S13, Supporting Information). This high degree of conservation suggests the potential for cross‐species application of therapeutic strategies targeting Hsp90β.

Hsp90β is an ATP‐dependent homodimeric chaperone protein consisting of three highly conserved domains: the N‐terminal domain (NTD; ≈25 kDa), the middle domain (M domain; ≈35 kDa), and the C‐terminal domain (CTD; ≈12 kDa).^[^ ^20^ ^]^ The N‐terminal domain harbors the ATP‐binding site, which also serves as a target for Hsp90 inhibitors. This domain has been extensively explored as a target for cancer therapy with drugs such as geldanamycin, its derivatives, and radicicol. These antitumor agents exhibit potent and specific inhibition of Hsp90 with nanomolar affinities.^[^ ^21^, ^22^, ^23^ ^]^ In this study, the N‐terminal domain of Hsp90β (N‐Hsp90β, amino acids 1–220) was produced and used to assess its binding capacity with Catalpol and Geldanamycin. DARTS, CETSA, and ITC assays revealed that N‐Hsp90β displayed binding affinities of ≈2.05 × 10^−6^ and 2.13 × 10^−6^ m for Catalpol and Geldanamycin, respectively. To gain further insight into the interaction between Catalpol and N‐Hsp90β, we used molecular docking and molecular dynamics simulation techniques. These techniques revealed the presence of water‐mediated hydrogen bonds between residues GLY92, ASN101, ASP49 of N‐Hsp90β and the hydroxyl groups of the sugar moiety in Catalpol. Furthermore, Catalpol displayed a significantly stronger binding affinity (−11.09 kcal mol^−1^) to N‐Hsp90β compared to the original ligand redocking result (−8.81 kcal mol^−1^). These findings suggest that water‐mediated hydrogen bonding plays a crucial role in the interaction of Catalpol with N‐Hsp90β, consistent with observations from previously crystallized protein complexes (PDB ID: 1UYM).^[^ ^24^, ^25^ ^]^ Intermolecular interaction analyses identified the hydroxyl group at the C6 position of the sugar moiety in Catalpol as a hydrogen bond donor with ASN46. RMSF analysis revealed that residues ASP88, GLY92, and THR179, located within the binding pocket, exhibited minimal fluctuations, suggesting their critical role in intermolecular interactions and protein conformation stabilization. Based on interaction statistics obtained during the 500 ns simulation, it was inferred that ASN46, ASP88, and THR179 played significant roles in forming a stable binding conformation. Collectively, these findings suggest that amino acids ASP88, THR179, ASP49, and ASN46 within the N‐terminal domain of Hsp90β may be critical for its interaction with Catalpol.

To investigate the mechanism by which Catalpol regulates OA progression, we performed transcriptomic profiling of OA chondrocytes and Catalpol treated OA cells by RNA sequencing. The result revealed that Catalpol treatment correlated with NF‐κB signaling pathway.^[^ ^26^ ^]^ By analyzing the transcriptome data of aged male OA chondrocytes and healthy male chondrocytes, it was found that there was a significant positive correlation between *Hsp90ab1* and *IKKα* and *Adamts4*, but a negative correlation between *Hsp90ab1* and *Bcl2*. In addition, *IKKα* was positively correlated with *Adamts4*, *IKKβ*, and *Bax*, while *Bax* was negatively correlated with *Sod1*, *Sox9*, and *Comp*. In addition, Hsp90 is known to regulate the enzymatic activity and biogenesis of IKKs, key molecules in the NF‐κB signaling pathway. Our results showed that inhibiting Hsp90β with either Catalpol or siRNA‐*Hsp90ab1* led to decreased expression of IKKα, IKKβ, and P65 and accompanied by increased expression of anabolic marker genes (*Col2a1* and *Acan*) and decreased expression of catabolic marker genes (*Mmp9*, *Mmp13*, and *Adamts4*). It is well‐established that NF‐κB signaling promotes the expression of inflammatory cytokines and catabolic factors in chondrocytes under oxidative stress or inflammatory conditions.^[^ ^27^ ^]^ Our findings support the notion that Catalpol acts as an inhibitor of Hsp90β, thereby regulating the anabolic and catabolic processes of chondrocytes through modulation of the NF‐κB pathway. This is consistent with previous reports demonstrating that Catalpol inhibits the inflammatory response and catabolism in IL‐1β‐induced rat chondrocytes via suppression of the NF‐κB signaling pathway.^[^ ^8^ ^]^

Intra‐articular administration remains the preferred approach for treating symptomatic OA. However, the limited potency of small‐molecule therapeutics hinders their efficacy due to rapid clearance and short residence time within the joint. To address this challenge, emerging nanocarriers such as MSNs are being investigated as effective drug delivery systems. Since their discovery in 2001, MSNs have shown promise in treating various diseases, including bone and tendon tissue engineering, diabetes, inflammation, and cancer.^[^ ^28^ ^]^ Studies have shown that MSNs loaded with Oltipraz [4‐methyl‐5‐(2‐pyrazinyl)‐1,2‐dithiole‐3‐thione (OL) exhibited chondroprotective potential after intra‐articular injection in OA models, with MSN‐OL persisting in the joint for at least 21 d.^[^ ^29^ ^]^ However, it is reported that prolonged nanoparticle accumulation may lead to inflammation, oxidative damage, and organ fibrosis, hindering clinical translation. In addition, MSNs with an average size of 25–100 nm exhibit a genotoxic effect in HT‐29 cells, whereas 30–300 nm do not demonstrate any harmful effects on the HeLa cells, suggesting that larger MSN particle sizes are associated with lower toxicity levels.^[^ ^30^ ^]^ Conversely, smaller MSNs are considered more toxic due to their increased cellular uptake and higher number of silanol groups interacting with the cell membrane. As a result, in this study, we developed degradable MSNs with an average size of 200 nm. These nanoparticles maintain drug release functionality while biodegrading and metabolizing rapidly within the body, thus avoiding inflammatory toxicity. Furthermore, we did not observe increased inflammation in the joint fluid of OA rats treated with bMSN or Ca‐bMSN by detecting inflammatory mediators (IL‐1β, IL‐6, and TNF‐α). It is noteworthy that the administration of bMSN alone may result in a modest amelioration of OA symptoms. This improvement may potentially be attributed to the nanoparticles’ capacity to inhibit inflammatory processes and establish an immune microenvironment conducive to bone regeneration, achieved through the activation of immune cells and the stimulation of cytokine release.^[^ ^31^ ^]^ In vitro studies demonstrated efficient uptake of bMSNs and Ca‐bMSN by mouse and human chondrocytes, and their ability to evade lysosomal degradation, suggesting excellent intracellular trafficking behavior. Furthermore, Ca‐bMSN promoted the expression of *Col2a1* and *Acan* while inhibited the expression of *Mmp13*, with no toxic effects on primary chondrocytes.

Fluorescent dyes are widely used to mark nanoparticles for fluorescence imaging in vivo, which usually have a limited half‐life. Meanwhile, MSNs have been reported to be as CT and optical imaging agents. Therefore, the in vivo degradation of bMSNs was assessed using micro‐CT, revealing almost complete degradation within 7 d, significantly reducing potential accumulation in the joint. Furthermore, HPLC analysis showed that bMSNs achieved sustained drug release compared to free Catalpol, thereby prolonging its duration of action. Following successful in vivo delivery, Ca‐bMSNs effectively suppressed the expression of MMP9, MMP13, and ADAMTS4 while increasing COL2A1 and ACAN levels in OA rat model, leading to improved OA symptoms. These findings demonstrate the potential of Ca‐bMSNs for OA treatment and provide an effective delivery and treatment strategy.

This study holds importance from multiple angles: (1) The identification of Catalpol as a promoter of chondrocyte proliferation and anabolism, along with the utilization of a delivery system for sustained release of Catalpol to chondrocytes, presents a novel approach for improving chondrocyte function and cartilage integrity. (2) Hsp90β was identified as a novel target of Catalpol, which enhances our comprehension of Catalpol's mechanisms of action and provides a basis for further exploration of the Catalpol/Hsp90β interaction in diverse conditions. The study acknowledges its limitations and suggests further research, including in vivo characterization and pharmaceutic kinetic assays of the drug in OA, as well as investigations with preclinical animal models and human clinical trials. While the study has shown that Hsp90β is the direct target of Catalpol, further exploration of the underlying mechanisms is warranted.

## 4 Conclusions 

In summary, this study demonstrates the potential of Catalpol as a therapeutic agent for OA. Our findings reveal that Catalpol stimulates chondrocyte anabolism, inhibits catabolism, oxidative stress, inflammation, and cell apoptosis, leading to reduced cartilage loss and alleviation of OA‐associated pain in vivo. In addition, we identify Hsp90β as a novel target of Catalpol, providing valuable insights into its mechanism of action and the underlying basis for its chondroprotective effects. Furthermore, the use of bMSNs as drug carriers enables prolonged and controlled release of Catalpol within the joint, significantly improving its in vivo efficacy. Overall, this study not only identifies an effective drug and target for promoting chondrocyte function and maintaining cartilage integrity but also presents a promising delivery strategy, contributing significantly to the development of targeted therapeutic candidates for OA.

## 5 Experimental Section

### Cell Culture

Primary chondrocytes were isolated from the rib cartilage of neonatal mice, as previously described. Briefly, the rib cartilage was infiltrated with 3 mg mL^−1^ collagenase B (Sigma, USA) for 45 min at 37 °C, followed by overnight digestion with 0.5 mg mL^−1^ collagenase B at 37 °C. The digestion was terminated with Dulbecco's Modified Eagle Medium/Ham's F12 (DMEM/F12) (Gibco, USA) supplemented with 5% fetal bovine serum (FBS) (BI, USA). The cells were collected by centrifugation (300 g, 5 min) and cultured in DMEM/F12 supplemented with 5% FBS (BI, USA) and 1% Penicillin/Streptomycin (Sigma, USA) at 37 °C and 5% CO_2_.^[^ ^32^ ^]^ Human chondrocyte cell line C28I2 was cultured in DMEM with 5% FBS, and 1% Penicillin/Streptomycin at 37 °C and 5% CO_2_.

### Quantitative Real‐Time Polymerase Chain Reaction (qRT‐PCR)

Total RNA was isolated from primary mouse chondrocytes, C28I2 cells, and cartilage tissues following the Total RNA Extraction Kit protocol (Tiangen, China). Subsequently, cDNA synthesis was carried out using the PrimeScript RT reagent Kit (TaKaRa, USA) with 1 µg of total RNA. qRT‐PCR was performed on the CFX96 Real‐Time System (Bio‐Rad, USA) utilizing the TB Green Premix Ex Taq II kit (TaKaRa, USA) along with specific primers (Table S1, Supporting Information). The relative gene expression level was determined using the 2^−ΔΔCT^ method, with Ribosomal Protein L4 (RPL4) serving as the internal control gene.

### CCK‐8 Analysis

The activity of small molecules was assessed using a combination of CCK‐8 assays. Briefly, primary mouse chondrocytes (5 × 10^3^ cells/well) were seeded in a 96‐well plate. Small molecules were individually added at final concentrations of 10, 20, 40, 60, 80, and 100 × 10^−6^ m to separate wells. The plates were then incubated for 24 h at 37 °C and 5% CO_2_. After incubation, 10 µL of CCK‐8 reagent (per well) was added and incubated for 1 h at 37 °C. Cell proliferation was measured by absorbance at 450 nm using a multifunctional microplate reader (Tecan, Switzerland).

### Western Blot Analysis

To prepare protein samples for Western blot analysis, chondrocytes or cartilage tissues were lysed using protein lysis buffer. The lysates were then loaded onto an SDS‐PAGE gel and electrophoretically transferred to a PVDF membrane (Millipore, USA). The membrane was blocked with 5% non‐fat milk in TBST for 2 h at room temperature. Primary antibodies (COL2A1, ACAN, MMP13, ADAMTS4, and RPL4; Proteintech, China) were incubated with the PVDF membrane overnight at 4 °C. Following washes, the membranes were incubated with the appropriate horseradish peroxidase (HRP)‐conjugated secondary antibodies for 1 h at room temperature. Protein bands were visualized using enhanced chemiluminescence (ECL) substrate (BeyoECL Plus, Beyotime, China) and detected with a ChemiDocXRS+ system (Bio‐Rad, USA). The dilutions for the primary antibodies were: COL2A1, ACAN, MMP13, and ADAMTS4 (1:1000) and RPL4 (1:2000).

### Immunofluorescent Assay

Cells were immobilized by incubation in 4% paraformaldehyde (Beyotime, China) for 10 min. Following three washes with PBS, cells were permeabilized with 0.5% Triton X‐100 (Solarbio, China) for 20 min. Subsequently, cells were incubated with anti‐COL2A1 antibodies (Proteintech, China; 1:500 dilution) for 2 h at 37 °C. To visualize the antigen–antibody complex, cells were treated with FITC‐labeled goat anti‐rabbit IgG (Proteintech, China; 1:1000 dilution) for 30 min. In addition, nuclei were stained with DAPI staining solution (Beyotime, China). Finally, images were captured using a cell imager (Thermo, USA).

### Alcian Blue Staining

OA chondrocytes were treated with varying concentrations of Catalpol (10, 20, and 40 × 10^−6^ m) for 7 d, the glycosaminoglycan content was quantified using an Alcian blue staining assay. Briefly, chondrocytes were stained with Alcian blue solution (Solarbio, China) for 2 h at room temperature, following the protocol described in a previous study. After staining, cells were washed, and the Alcian blue dye was extracted with 6 m guanidine hydrochloride (Macklin, China). The amount of extracted dye was quantified by measuring the optical density (OD) at 620 nm using a multifunctional microplate reader.^[^ ^33^ ^]^

### Evaluation of ROS Levels

To assess the level of reactive oxygen species within chondrocytes, the Reactive Oxygen Species Detection Kit (Beyotime, China) was used following the manufacturer's instructions. Briefly, cells were incubated with the kit's probes for 20 min at 37 °C. Following incubation, fluorescence intensity was measured using a flow cytometer (Beckman Coulter, USA) to quantify ROS levels.

### Analysis of SOD, MDA, GSSG, and ATP Level

Primary chondrocytes were treated with IL‐1β alone or in combination with Catalpol (10, 20, or 40 × 10^−6^ m) for 24 h. After that, cells were washed with precooled PBS and lysed. The supernatant was obtained by centrifuging and used for SOD, MDA, GSSG, total antioxidant, and ATP detection using specific assay kits (Beyotime, China) according to the manufacturer's protocol.

### Mitochondrial Membrane Potential Determination

Primary chondrocytes were treated with IL‐1β and IL‐1β + Catalpol (10, 20, 40 × 10^−6^ m). After 24 h of culture, mitochondrial membrane potential was detected by Mitochondrial membrane potential assay kit with JC‐1 (Beyotime, China) according to the experimental protocol. In detail, 0.3 mL of JC‐1 staining solution was added to each well of 24‐well plate, and incubated in cell incubator at 37 °C for 20 min. Images were obtained by cell imager. Quantification was performed on a multifunctional microplate reader.

### Analysis of Cell Apoptosis

Mouse primary chondrocytes were divided into control, model (cells treated with 10 ng µL^−1^ IL‐1β) and Catalpol (10, 20, 40 × 10^−6^ m + 10 ng µL^−1^ IL‐1β) groups. After treatment with IL‐1β or IL‐1β + Catalpol, chondrocytes were prepared by incubation with 300 µL binding buffer including 5 µL Annexin V, followed by 5 µL propidium iodide (PI) for 5 min at 37 °C in the dark. Analysis was conducted using a flow cytometer.

### RNA Sequencing and Data Analysis

Mouse primary chondrocytes were divided into control, model (cells treated with 10 ng µL^−1^ IL‐1β) and Catalpol (20 × 10^−6^ m + 10 ng µL^−1^ IL‐1β) groups and processed for RNA extraction. The RNA sequencing and data analysis were performed by Novogene Co., Ltd. In addition, differential expression analysis in human chondrocytes RNA sequencing data (GEO: GSE 246425) was performed with Voom and Limma methods (Limma package). The GO annotations and correlation between target genes and were conducted via a free online platform for data visualization and graphing (https://www.bioinformatics.com.cn).

### Preparation of bMSN and bMSN‐FITC

The synthesis of bMSNs was performed using a modified procedure. A three‐necked flask was utilized to stir 1.2 g of cetyl trimethyl ammonium bromide (CTAB), 0.30 g of triethanolamine (TEA), and 100 mL of deionized water at 80 °C for a duration of 30 min. Subsequently, a solution containing 1800 µL of TEOS and 200 µL BTES was gradually added to the surfactant solution over a period of ≈0.5 h.

The resulting mixture was then stirred at 80 °C for an additional 4 h at a stirring speed of 1000 rpm. The products were obtained through centrifugation (11000 rpm, 5 min), followed by three washes with ethanol. Finally, the collected products were refluxed in an ethanol solution of HCl (2% w/v) for a duration of 6 h. The bMSNs were collected, washed, and dried. To attach amine groups to the MSNs, 20 mg of MSNs was suspended in 20 mL of dimethylformamide. 10 µL of 3‐aminopropyl triethoxysilane (APTES) was added and stirred for 24 h at room temperature. The resulting MSN‐NH_2_ was then washed three times with EtOH to remove any remaining APTES and dried. For the fluorescent modification of bMSN, 0.5 mg of FITC was added to 10 mL of bMSN‐NH_2_ solution (1 mg mL^−1^ in absolute ethanol). The mixture was stirred overnight in the dark. The FITC‐modified bMSNs were collected via centrifugation and subsequently washed using ethanol.

### Physicochemical and Ex Vivo Characterization of Nanoparticles

The morphology and structure of MSNs were examined using a TEM (Hitachi HT‐7700, Japan). The TEM was operated at 100 kV. The particle size and zeta potential were assessed using Zetasizer (Malvern, UK).^[^ ^34^ ^]^ To analyze the drug loading efficiency of bMSNs, the wetness impregnation method was used to load Catalpol into the bMSNs. Briefly, 1 mL solution of Catalpol (1 mg mL^−1^) was added into deionized water containing 1 mg of bMSNs with gentle continuous stirring and ultrasonic mixing for 24 h, then centrifuged at 11000 rpm, 5 min at 4 °C. The supernatant was finally collected. The Catalpol drug content in the loaded MSNs was determined using Agilent 1260 HPLC with monitoring of Catalpol absorbance at 203 nm.^[^ ^35^ ^]^ The encapsulation efficiency (EE%) was calculated as follows: EE (%) = (Weight of Catalpol in loaded MSNs/Weight of feeding Catalpol) × 100. The weight of Catalpol in loaded MSNs was determined by subtracting the weight of Catalpol in the supernatant from the weight of feeding Catalpol. For analysis of the drug stability and release in the synovial fluid, the rats were anesthetized and the synovial fluid was taken from articular joint. Synovial fluid was divided into two groups, and Catalpol (20 × 10^−6^ m) and Ca‐bMSNs (10 mg mL^−1^) were added, respectively. Samples were gently mixed in the shaker at 37 °C, and then collected at predetermined time intervals (0, 1, 3, 12, and 24 h). Concentrations in the release medium were determined by HPLC.

### Absorption and Degradation of bMSNs and Ca‐bMSNs In Vitro and In Vivo

Primary mouse chondrocytes and human chondrocytes (C28I2) were used for investigating the cellular uptake of bMSNs and Ca‐bMSNs. Briefly, chondrocytes were incubated in glass bottom cell culture dish at a density of 5 × 10^5^ cells per well and cultured for 1 d. The bMSNs and Ca‐bMSNs were labelled with FITC and then added into plates at a concentration of 10 µg mL^−1^ to assess intracellular efficiency. After incubation of chondrocytes with bMSNs or Ca‐bMSNs for 4 h, the cell samples were washed three times with PBS. Then, the cells were stained with Hoechst 33342 (Beyotime, China) for 20 min at 37 °C to label nuclei. The images were captured and analyzed by laser confocal microscopy (Leica Germany).^[^ ^36^ ^]^ The degradation of bMSNs and Ca‐bMSNs in rats joint was detected by Micro‐CT (Perkin Elmer, USA) after receiving an IA injection of 100 µL bMSNs and Ca‐bMSNs (200 µg mL^−1^) at days 0 (after IA injection immediately), 1, 3, 5, and 7.

### Hemolytic Assays

Blood was collected from the abdominal aorta and preserved in anticoagulant tubes for the isolation of red blood cells (RBCs). The RBCs underwent five washes with a PBS solution, followed by centrifugation at 4000 rpm for 10 min at 4 °C to remove the supernatant. A 5% stock suspension of RBCs in PBS was then prepared for use in hemolytic assays. Ca‐bMSNs and bMSNs (1, 5, 10, 100 mg mL^−1^) were incubated with 500 µL of the RBC stock suspension for 4 h at room temperature. Following incubation, the tubes were centrifuged for 10 min, and the supernatants were carefully transferred to a clean 96‐well plate for detection using a multifunctional microplate reader set at 485–700 nm. ddH_2_O_2_ served as positive control. Saline was used with negative control. The percentage of hemolysis = [optical density − negative control optical density/(positive control optical density − negative control optical density)*100.

### Intracellular Trafficking of bMSNs and Ca‐bMSNs in Chondrocytes

To demonstrate whether bMSNs and Ca‐bMSNs could avoid lysosomal elimination in chondrocytes, chondrocytes were seeded at a density of 5 × 10^5^ cells per dish and cultured for 24 h. The medium was replaced with fresh medium containing bMSNs or Ca‐bMSNs (labeled with FITC) at a concentration of 10 µg mL^−1^ and incubated for an additional 4 h. Subsequently, the lysosomes were labeled with LysoTracker Green (200 × 10^−9^ m) for 30 min. In addition, the nuclei were stained with Hoechst 33342 for 20 min. The images were then acquired and examined using laser confocal microscopy.^[^ ^36^ ^]^

### Establishment of OA Model and Administration of Drugs

The animal protocol of this study was approved by the Institutional Animal Care and Use Committee of Changchun University of Chinese Medicine (No. 2023029). Adult male Sprague‐Dawley (SD) rats (8‐weeks‐old) were randomly divided into sham group (*n* = 10), model group (*n* = 10), bMSN group (10 mg mL^−1^, *n* = 10), Catalpol group (20 × 10^−6^ m, *n* = 10), Ca‐bMSN group (1, 5, 10 mg mL^−1^, *n* = 10, 10, 10), and HA group (10 mg mL^−1^, *n* = 10). The OA model was made through destabilization of the medial meniscus (DMM) combined with running motion (25 m min^−1^, 80 min) (Xinruan, China) for four weeks.^[^ ^37^, ^38^ ^]^ Rats with OA were injected with different components (bMSN, Catalpol, bMSN + Catalpol) in the knee joint cavity weekly (100 µL/time). Adult male C57BL/6 mice (8‐weeks‐old) were randomly divided into sham group (*n* = 10), model group (*n* = 10), bMSN group (10 mg mL^−1^, *n* = 10), Catalpol group (20 × 10^−6^ m, *n* = 10), Ca‐bMSN group (1, 5, 10 mg mL^−1^, *n* = 10, 10, 10), and HA group (10 mg mL^−1^, *n* = 10). Rats and mice were sacrificed after four weeks of continuous treatment administration.

### Behavioral Assessment

Gait analysis of rats in the sham group, model group, bMSN group, Catalpol group, and Ca‐bMSN group was performed on gait analyzer (Mouse Specifics Inc., USA). The rats were placed on a transparent conveyor belt at a running speed of 20 m s^−1^. DigiGait video imaging acquisition system (Mouse Specifics Inc., USA) was used to observe each paw area (mm^2^), weight (%), posed duration (s), and stride length (cm).

### Micro‐CT, Histology, and IHC

The complete knee joint of the rats and mice in the different group was scanned using a micro‐CT system (Perkin Elmer, USA). The surface smoothness of cartilage and the degree of subchondral bone sclerosis were detected in coronal and sagittal positions. Reconstruction of 3D images was obtained with N‐Recon software, BMD, BV/TV, BS/TV, and Tb.Th were measured by Analyze 12.0 software (AnalyzeDirect, Inc., USA).^[^ ^32^ ^]^ For histology and IHC analysis, knee joints were embedded in wax blocks followed by fixed in 4% paraformaldehyde and then were sectioned at 4 µm thickness. Each section was stained with H&E and safranin‐O‐fast green to observe the cartilage, or antibodies including Col2a1, Acan, Mmp13, and Adamts4 to detect specific protein expression level. The images were captured using a digital scanning microimaging system M8 (Precipoint, Germany).

### Drug Affinity Responsive Target Stability Assay and Mass Spectrometry

The DARTS assay was carried out according to previous reports.^[^ ^39^, ^40^ ^]^ In brief, total protein was obtained from mouse primary chondrocytes that lysed with RIPA lysis buffer (Beyotime, China), then centrifuged at 12000 rpm. 50 µL of total protein was incubated with PBS or Catalpol (500 × 10^−6^ m) for 20 min at 25 °C. The mixture was digested by protease (Sigma, USA, 1:100, 1:200) for 30 or 40 min. Samples were loaded on SDS‐PAGE, and stained with silver to detect target protein band. Target band was then analyzed by mass spectrometry performed by Novogene Co., Ltd.

### Expression and Purification of Full Length and Truncated Hsp90β Protein

Full‐length Hsp90β and its N‐terminal domain (N‐Hsp90β, residues 1–200) were cloned into the pET‐28a (+) vector, each with an N‐terminal His‐tag. The constructs were then transformed into *Escherichia coli* BL21 (DE3) cells (Tiangen, China). Single colonies were grown in culture at 37 °C with shaking at 200 rpm until the OD600 reached 0.6. Protein expression was induced by adding 1 × 10^−3^ m isopropyl β‐d‐thiogalactoside (IPTG) and incubating the cells for 24 h at 16 °C with shaking at 180 rpm. Following induction, cells were harvested by centrifugation at 4000 rpm, resuspended in PBS, and lysed by sonication for 1 h (50% power, 2 s on, 3 s off) using a sonicator (Scientz, China). Cell debris was removed by centrifugation at 12000 rpm. The supernatant was loaded onto a nickel affinity column (Cytiva, Sweden) and washed with 100 mL of washing buffer (PBS with 10 × 10^−3^ m imidazole) to remove unbound proteins. His‐tagged Hsp90β or N‐Hsp90β protein was then eluted with elution buffer (PBS with 250 × 10^−3^ m imidazole). The eluted proteins were dialyzed against PBS and concentrated using ultrafiltration with a 10 kDa molecular weight cutoff membrane (Millipore, USA). Protein purity was assessed by SDS‐PAGE with Coomassie Brilliant Blue staining.^[^ ^41^ ^]^

### Cellular Thermal Shift Assay

The mouse primary chondrocytes lysates were diluted with PBS and divided into two groups, with one group treated with PBS, another aliquot with Catalpol (500 × 10^−6^ m). After 20 min incubation at 25 °C, the lysates were divided into 20 µL/tube and heated individually at gradient temperatures (46–79 °C) for 3 min followed by cooling for 3 min at 4 °C. Samples were detected by immunoblot with Hsp90β antibodies (Selleck, USA, 1:1000) or His‐Tag antibodies (Proteintech, China, 1:1000).^[^ ^42^, ^43^, ^44^ ^]^

### Isothermal Titration Calorimetry Analysis

ITC analysis was conducted at 25 °C, utilizing the MicroCal PEAQ‐ITC instrument (Malvern, Sweden) with the titration of 13 injections of 2 µL of Catapol (200 × 10^−6^ m) or Geldanamycin into the reaction cell containing 20 × 10^−6^ m of purified full length Hsp90β or N‐Hsp90β protein solution. The Catapol and protein were prepared with same buffer. Titration of Catapol to the same buffer was used as control to calibrate the equipment. Binding isotherms were fitted using nonlinear regression with the MicroCal PEAQ‐ITC analysis software.

### Ligand, Protein Preparation, and Receptor Grid Generation

The 3D molecule model of Catalpol was constructed and geometrically optimized using ChemSketch. LigPrep was further used to ionize the ligand based on the Epik module. The Hsp90β N‐terminal domain (PDB ID: 5uc4) underwent processing via Protein Preparation Wizard, which is part of the Schrodinger Suite in Maestro.^[^ ^25^ ^]^ The water molecules that were not involved in ligand interactions and were situated at a distance greater than 5 Å from the ligand were eliminated from the original protein crystal structure. The missing loop or side chains in the crystal structure were reconstructed using Prime. Subsequently, hydrogen bonds were established with all amino acid residues at pH 7.0, considering their least ionized states. Energy minimization was performed using the OPLS‐3 force field until a RMSD of 0.30 was attained, effectively reducing steric hindrance. The grid box was created to characterize the centroid of the binding site for docking purposes. The box is generated using the Glide grid generation wizard, and its size was set to match that of the crystallized ligand.

### Molecular Docking and Binding Free Energy Calculations Based on MMGBSA Method

After optimizing Catalpol using LigPrep, it was subjected to docking within a recently established grid box using Glide's extra‐precision mode, without any constraints.^[^ ^45^ ^]^ To overcome the steric hindrance, the OPLS‐3 force field was utilized to minimize energy until a RMSD of 0.30 was attained. The activate site was formed by applying the coordinates of preexisting co‐crystals protein–ligand complex (PDB ID: 5uc4), while a centroid grid box was used to create the binding pocket. The resulting grid box was used for docking Catalpol in Glide's “extra‐precision mode.” Subsequently, the docking pose of Catalpol was selected for the computation of Gibbs free energy, using the OPLS‐3 force field, the VSGB solvent model, and the rotamer search tools.

### Molecular Dynamic Simulation

A 500‐ns MD simulation was performed to assess the stability of the Catalpol–Hsp90β complex after docking. To investigate this complex in an explicit solvent system, the Desmond module of the Schrodinger suite was used, applying the OPLS3 force field. The molecular system underwent solvation with the TIP3P model and was subjected to truncated octahedral periodic boundary conditions. The elimination of overlapping water molecules and neutralization of the system were accomplished through the introduction of sodium counterions. The system consists of 24183 atoms and 6947 water molecules. Stable conditions were ensured at 300 K and 1 bar using a Nose–Hoover thermostat and barostat ensemble (*NPT*). An energy minimization method, consisting of a hybrid technique involving 1000 steps of steepest descent and the Broyden‐Fletcher Goldfarb‐Shanno (LBFGS) algorithm, was used. The Smooth Particle Mesh Ewald method was used to facilitate long‐range electrostatic interactions, while short‐range van der Waals and Coulomb interactions were limited to a cut‐off radius of 9 Å. The dynamics of bonded, near‐bonded, and far‐bonded interactions was examined using multiple the time step RESPA integration (reference system propagator approaches) at intervals of 2, 2, and 6 frames s^−1^, respectively. Data were collected every 200 ps, and the computational trajectory was assessed through simulation event analysis.

### Silencing of *Hsp90ab1* in Primary Chondrocytes and in OA Rats

For the experiment of *Hsp90ab1* silencing in chondrocytes, the incubation mixture comprising 10 pmol *Hsp90ab1* silencer (Genepharma, China) or negative control siRNA (Genepharma, China), 2 µL Lipofectamine (Thermo, USA) and 47 µL OPTI medium (Gibco, USA) was added to a 24‐well plate, and each well contained 1 × 10^5^ cells growing without adherence. The cells were grown in a 5% CO_2_, 95% atmospheric air incubator at 37 °C. For the experiment of *Hsp90ab1* silencing in OA rats, siRNA‐*Hsp90ab1* was modified by ago for intra‐articular injection in rats. The sequence of ago‐siRNA‐Hsp90ab1 or negative control siRNA was designed and constructed by GenePharma Co., Ltd. 6 nmol siRNA‐*Hsp90ab1* in 10 µL PBS were injected into OA rat joint cavity. OA rats received injections once weekly for four weeks.^[^ ^46^ ^]^ Silencing efficiency was then detected by qRT‐PCR using specific primers listed in Table S1 (Supporting Information). The information of siRNA is listed in Table S2 (Supporting Information).

### Protein–Protein Interaction (PPI) Network

Protein–protein interaction network was constructed and downloaded from the STRING database (https://www.string‐db.org/).

### Statistical Analysis

The numerical data presented in the figures were analyzed by SPSS software and presented as the mean ± SEM (*n* ≥ 3). Significant differences between the two groups were determined to use unpaired Student's *t*‐tests. ANOVA was used for multiple comparisons. The data were tested for normality of distribution using Shapiro–Wilk test before analysis. *P*‐values < 0.05 were considered statistically significant.

## Conflict of Interest

The authors declare no conflict of interest.

## Ethical Statement

This study was approved by the Ethics Committee of Changchun University of Traditional Chinese Medicine (No. 2023029).

## Author Contributions

Designed and drafted the manuscript: H.D., H.S., Z.Z., X.L., and D.Z. Methodology: Z.Z., H.S., H.D., and X.L. Chondrogenesis‐related experiments: B.Z., L.L., J.Y., and Z.Z. Identified the drug target: Z.Z., H.D., B.Z., and L.L. Drug screening: H.S., Y.W., and Z.Z. Synthesis and characterization of bMSNs and Ca‐ bMSNs: C.L. and Y.W. Micro‐CT analysis: J.Y. and H.Z. Expression and purification of protein: Y.W., Z.J., and Z.P.

## Supporting information



Supporting Information

## Data Availability

The data that support the findings of this study are available from the corresponding author upon reasonable request.
