# Supplementary material for: Inhibition of Heat Shock Protein 90β by Catalpol: A Potential Therapeutic Approach for Alleviating Inflammation‐Induced Cartilage Injuries in Osteoarthritis
Source: Adv Sci (Weinh). 2025 Apr 25;12(26):2503909. doi: 10.1002/advs.202503909 (PMC12245135; doi:10.1002/advs.202503909)

Supporting Information

**Inhibition of Heat Shock Protein 90β by Catalpol: A Potential Therapeutic Approach for Alleviating Inflammation-Induced Cartilage Injuries in Osteoarthritis**

*Zhenwei Zhou^1,3^, Binghua Zhang^2^, Lang Liu^2^, Jie Yang^2^, Yuting Wang^1^, Cheng Lv^1^, He Zhang^1^, Yuchi Wei^2^, Zhanliang Jiang^2^, Zeyu Peng^2^, Daqing Zhao^1^*, Xiangyang Leng^2^*, Xiangyan Li^1^*, Hang Su^1^*, Haisi Dong^1,3^**

**Affiliations**

1 Affiliated Hospital of Changchun University of Traditional Chinese Medicine, Changchun University of Chinese Medicine, Changchun 130000, Jilin Province, China.

2 College of Traditional Chinese Medicine, Changchun University of Chinese Medicine, Changchun 130000, Jilin Province, China.

3 Northeast Asia Institute Research of Traditional Chinese Medicine, Changchun University of Chinese Medicine, Changchun 130000, Jilin Province, China.

^*^Correspondence: Xiangyang Leng, Daqing Zhao, Xiangyan Li, Hang Su and Haisi Dong. Email: zhaodq@ccucm.edu.cn; lengxy@ccucm.edu.cn; lixy01@ccucm.edu.cn; suhang01@ccucm.edu.cn; donghs@ccucm.edu.cn; Address：No.1035, Boshuo Road, Changchun 130000, Jilin Province, China.


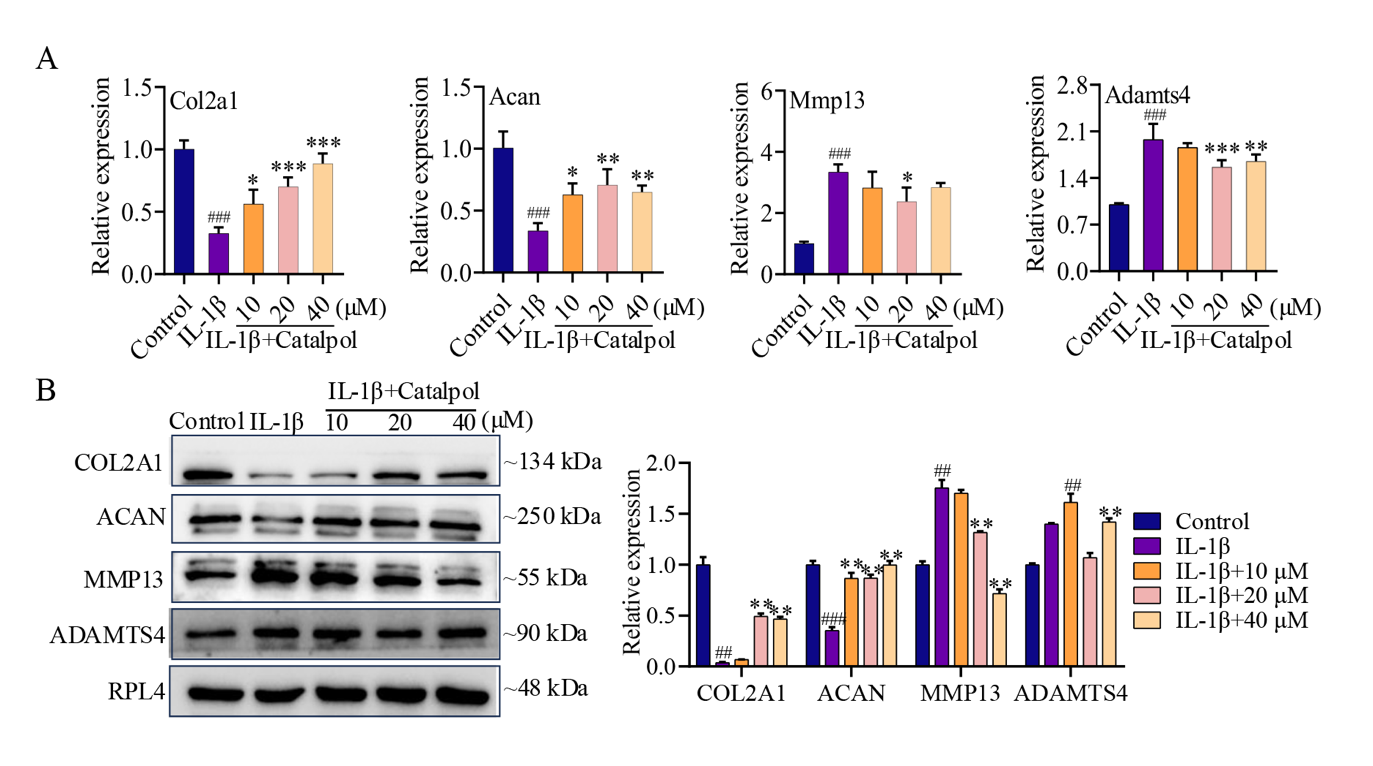


**Figure S1. The effect of Catalpol on human chondrocytes anabolism and catabolism.** (A-B) qRT-PCR (A) and Western blot (B) analysis of the expression level of anabolism marker genes (*Col2a1, Acan*) and catabolism marker genes (*Mmp13, Adamts4*) in human chondrocytes treated with IL-1β or IL-1β combined with different concentrations of Catalpol at 24 h. The quantification result was shown at right. n=3, # *vs* Control, * *vs* IL-1β, * p < 0.05, ## or ** p < 0.01, ### or *** p < 0.001. Data are shown as mean ± SEM. P values were obtained by one-way ANOVA with multiple comparisons.


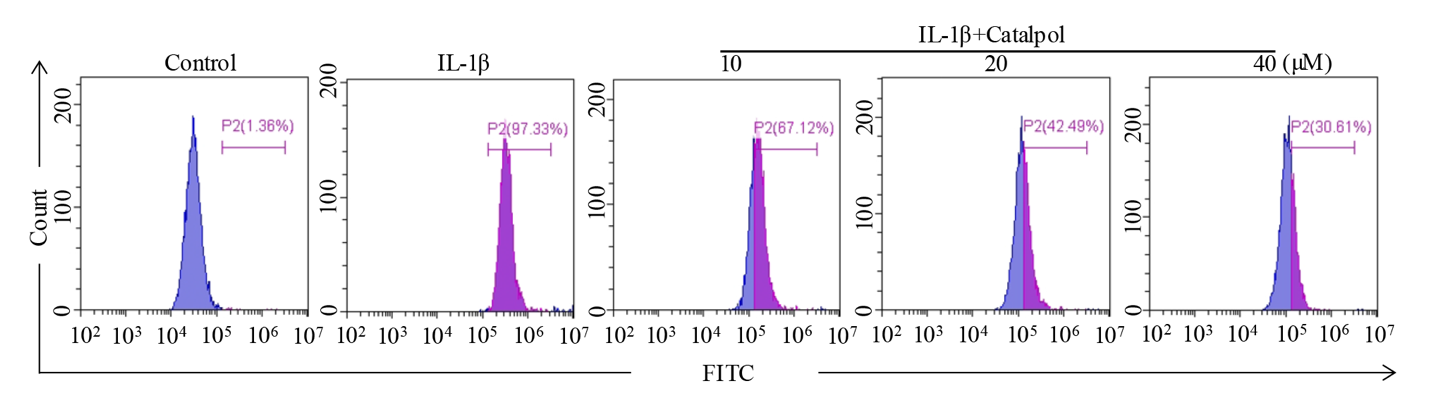


**Figure S2. Catalpol reduced ROS production in human chondrocytes induced by IL-1β.** Human chondrocytes were treated with IL-1β or IL-1β combined with different concentrations of Catalpol at 24 h, ROS production in human chondrocytes was determined by flow cytometric analysis.


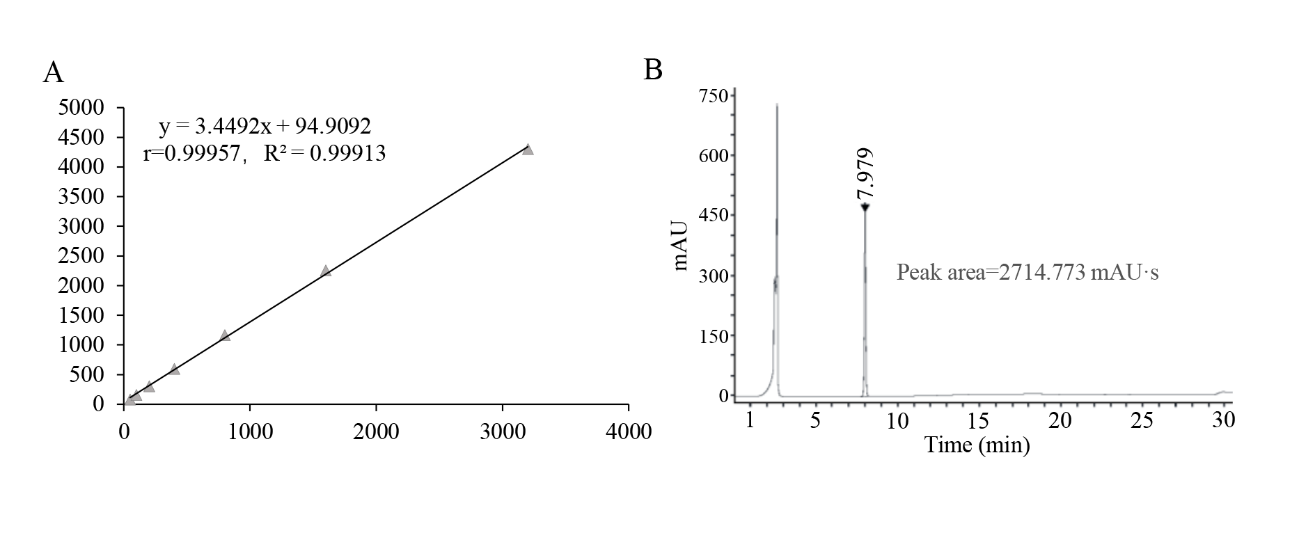


**Figure S3. The Catalpol loading efficiency of bMSN.** (A) The HPLC standard curve of Catalpol. (B) The peak area of Catalpol in HPLC chromatograms.


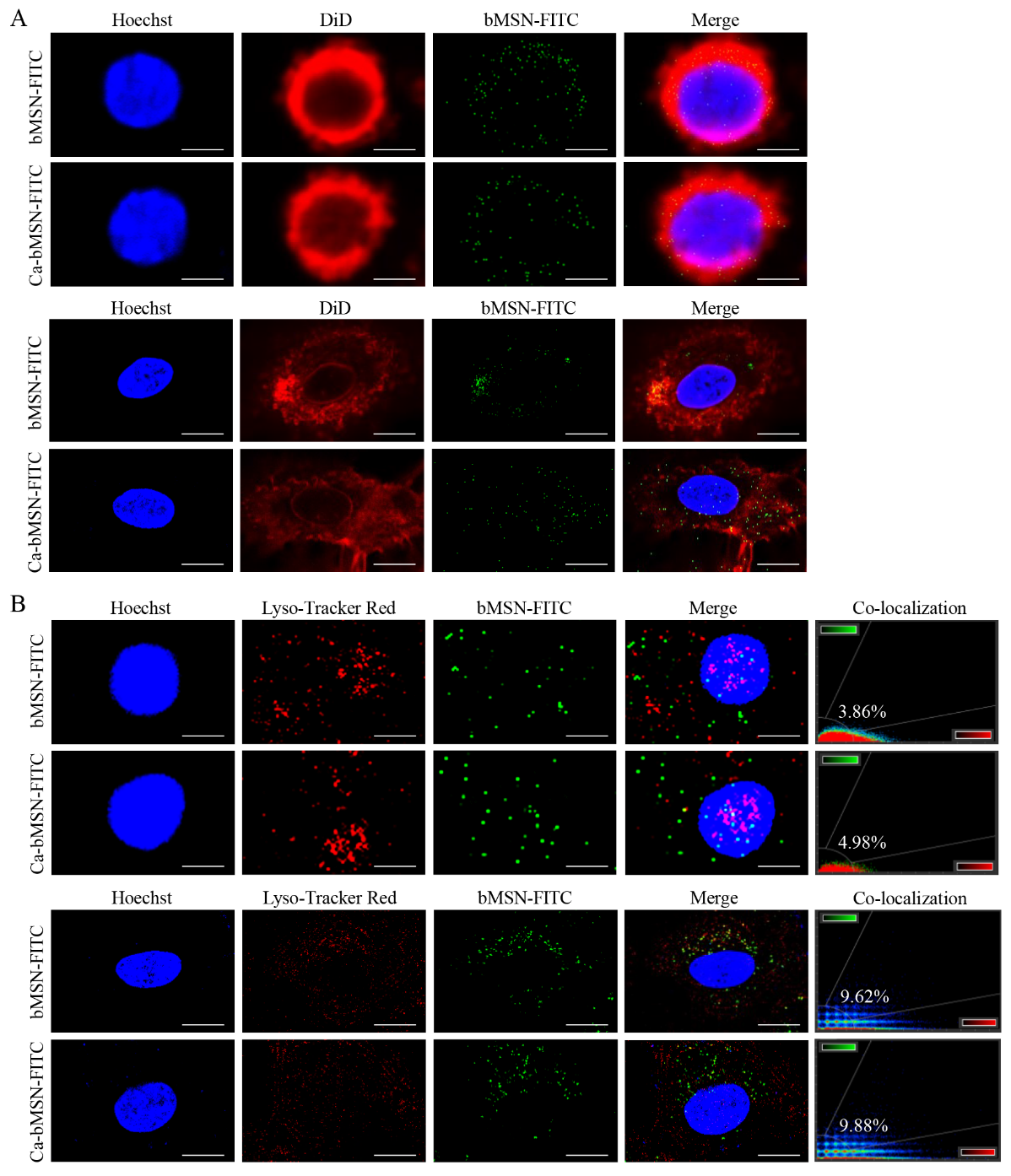


**Figure S4. The transport of bMSN and Ca-bMSN in mouse and human chondrocytes.** (A) Uptake of bMSN and Ca-bMSN by mouse (top) and human (bottom) primary chondrocytes. Nuclei, blue; Cell membrane, red. Scale bar = 5 µm. (B) Fluorescent visualization co-localization between bMSN or Ca-bMSN and lysosome in mouse (top) and human (bottom) primary chondrocytes. bMSN or Ca-bMSN, green; Nuclei, blue; Lysosome, red. The quantification result was shown at right. Scale bar = 5 µm.


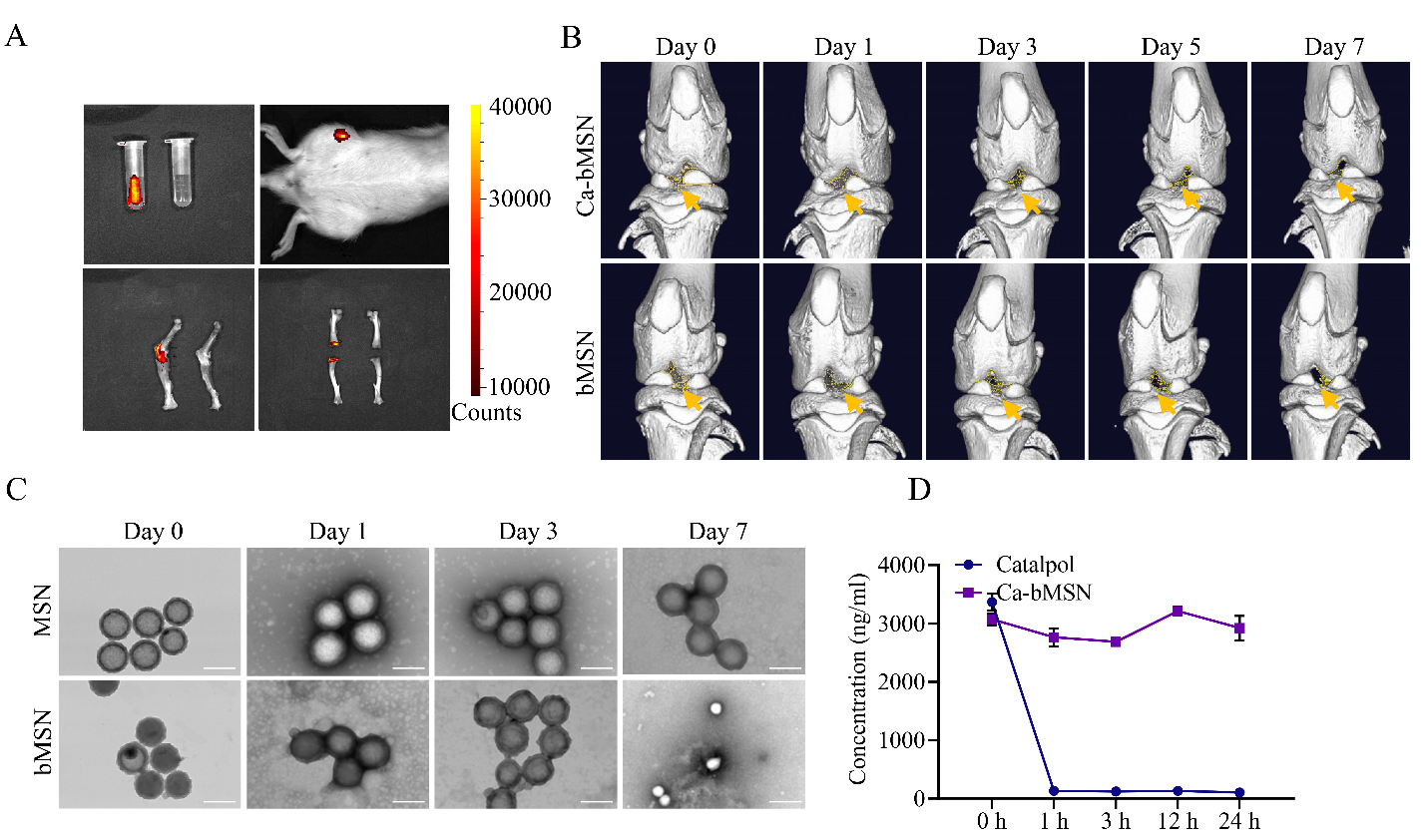


**Figure S5. The retention of bMSN and Ca-bMSN *in vivo*.** (A-B) Fluorescence imaging and Micro-CT were used to observe the bMSN and Ca-bMSN degradation in the knee joint cavity, bMSNs and Ca-bMSNs are marked by yellow and yellow arrows, (n = 5). (C) TEM images showing the biodegradation behavior of MSN and bMSN at various time points. Scale bar = 200 nm. (D) Comparative evaluation of the drug release of Catalpol alone and Catalpol-loaded bMSN detected by HPLC.


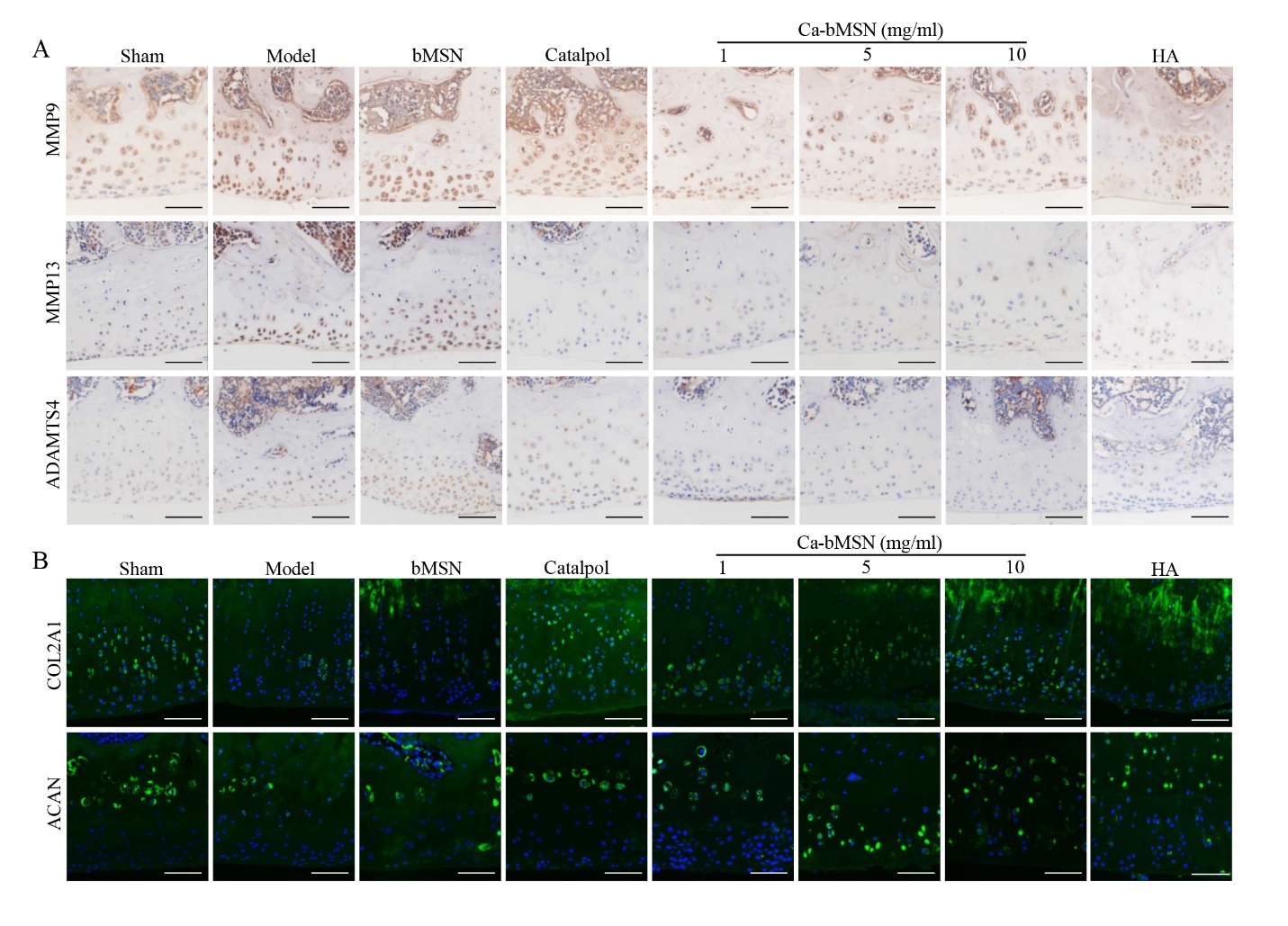


**Figure S6. The effect of Ca-bMSN on chondrocytes anabolism and catabolism *in vivo*.** The protein expression levels of MMP9, MMP13, ADAMTS4, COL2A1 and ACAN in knee cartilage tissues were measured by immunohistochemical (A) and immunofluorescent staining (B). Scale bar = 100 µm.

**
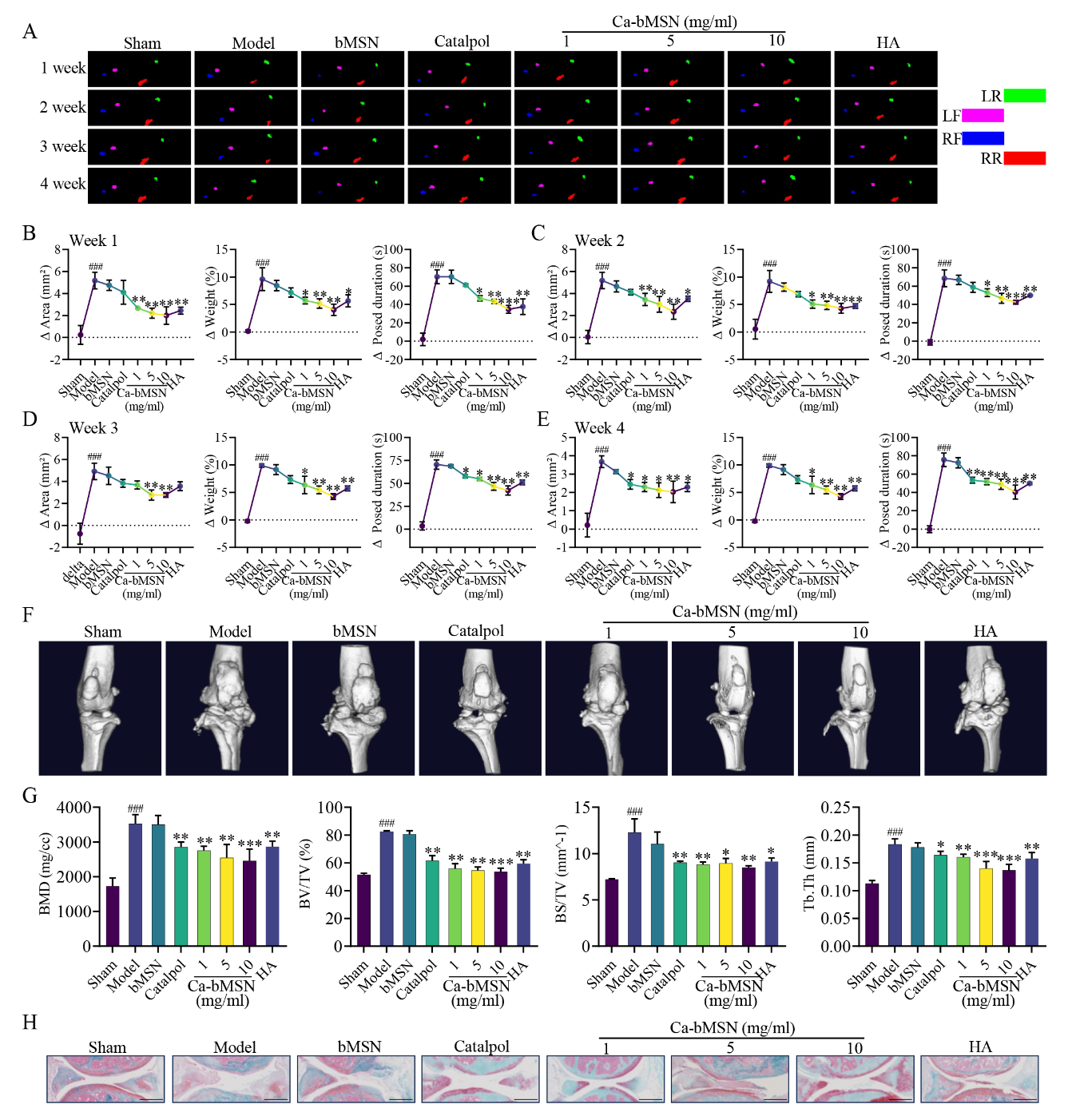
**

**Figure S7. *In vivo* effects of** **Catalpol, bMSN and Ca-bMSN treatment on OA mouse model.** (A) Gait analysis images of Sham, Model, bMSN, Catalpol, and Ca-bMSN groups. LF, left front paw; RF, right front paw; LR, left rear paw; RR, right rear paw. (B-E) Δ Area, Δ Weight and Δ Posed duration of sham, model Catalpol, bMSN and Ca-bMSN groups at week 1, week 2, week 3 and week 4. (n = 5, * p < 0.05, **p < 0.01, ### or *** p < 0.001 when compared with sham group and model group, respectively). (F) Three-dimensional images of the knee joints by micro-CT at 4 weeks of treatment with Catalpol, bMSN and Ca-bMSN. Arrows indicate the lesion points of joint. (G) Quantitative analysis of BMD, BV/TV, BS/TV and Tb.Th. (n = 5, * p < 0.05, ** p < 0.01, ### or *** p < 0.001 when compared with sham group and model group, respectively). (H) Safranin-O-fast green staining (SO/FG) of knee joints treated with Catalpol, bMSN and Ca-bMSN at 4 weeks. Scale bar = 500 µm. Data are shown as mean ± SEM. P values were obtained by one-way ANOVA with multiple comparisons.


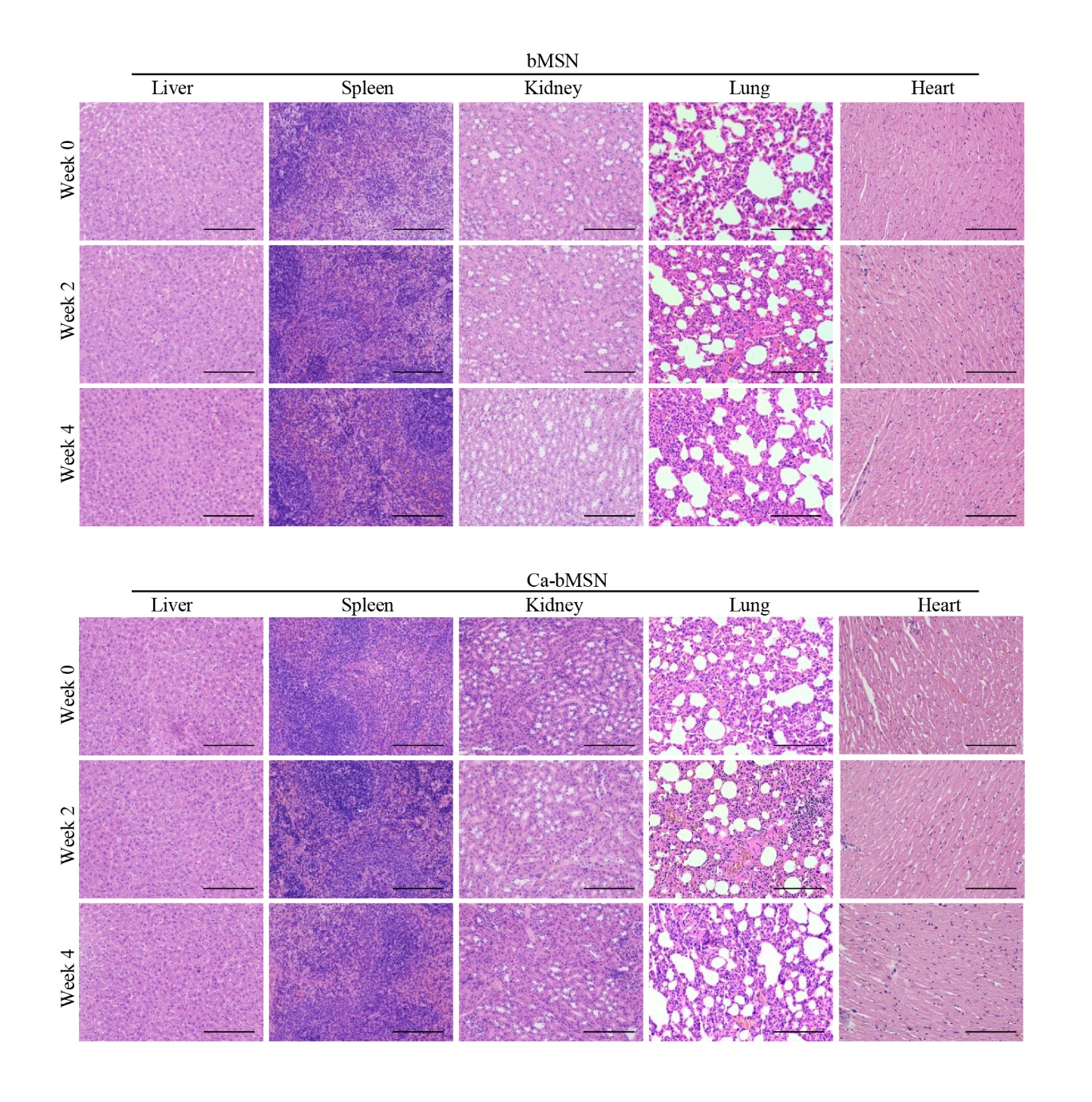


**Figure S8. Histological evaluation of systemic toxicity *in vivo*.** Representative images of H&E staining of rat liver, spleen, kidney, lung and heart after injections of bMSN and Ca-bMSN at 0, 2, 4 weeks. Scale bar = 200 μm.


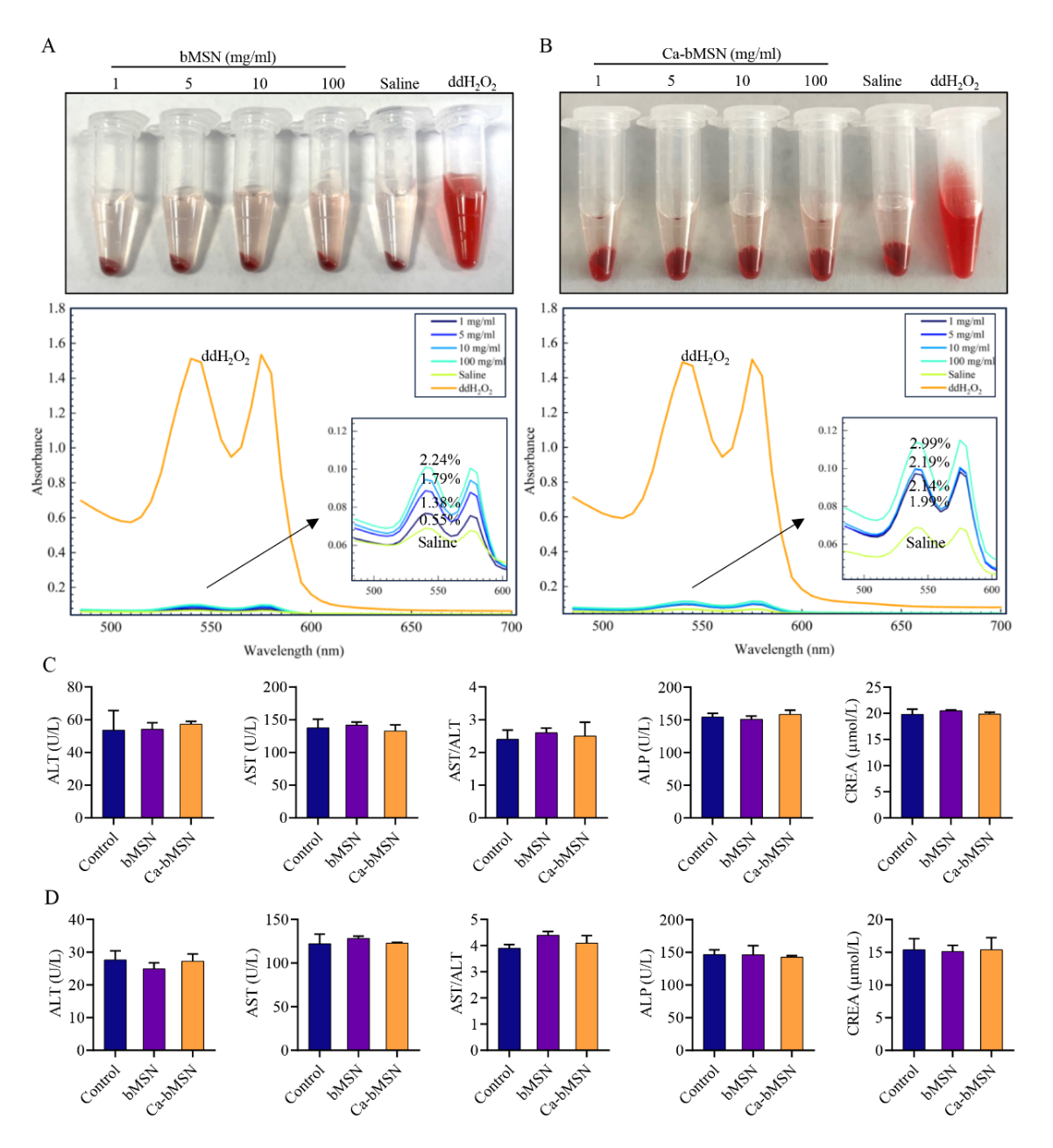


**Figure S9. The toxicity of the bMSN and Ca-bMSN was checked using a hemolytic assay and** **an automatic biochemical instrument.** (A-B) The hemolysis percentage of bMSN and Ca-bMSN, ddH_2_O_2_ served as positive control and saline as a negative control. (C-D) The level of ALT, AST, AST/ALT, ALP and CREA, (C) rat, (D) mouse.


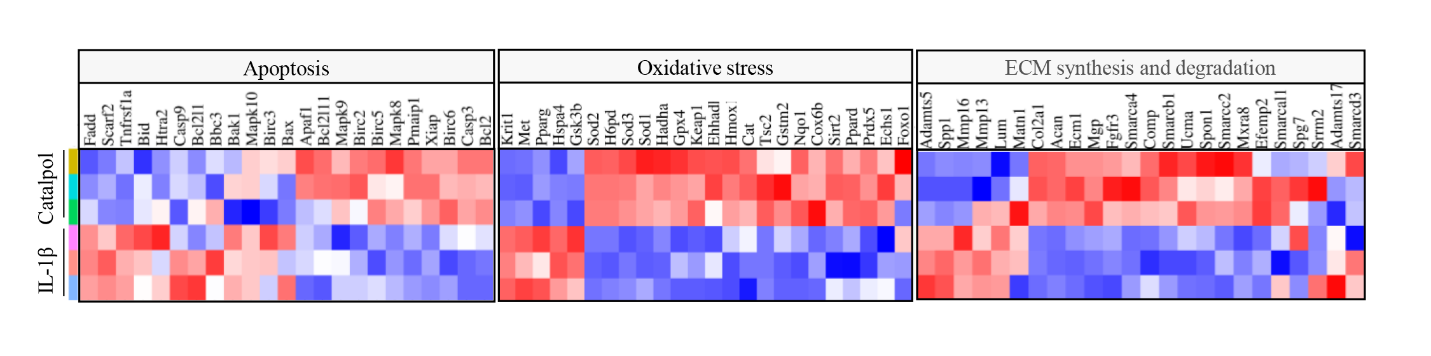


**Figure S10. Heat map of gene expression associated with chondrocytes apoptosis, oxidative stress and matrix catabolism.**

**
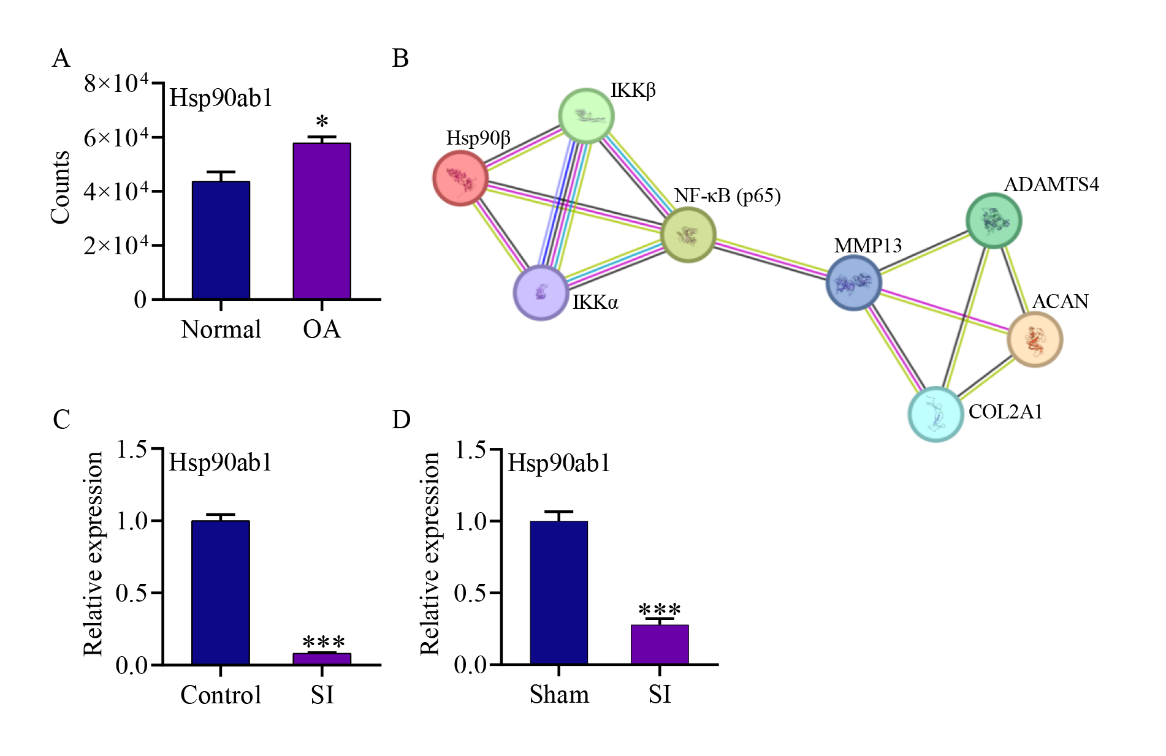
**

**Figure S11.** **The expression level of *Hsp90ab1* gene and analysis of protein-protein interactions.** (A) *Hsp90ab1* gene read counts in the health male chondrocytes and OA male chondrocytes. (B) Protein-protein interactions among the Hsp90β, IKKβ, IKKα, NF-κB (p65), MMP13, ADAMTS4, COL2A1 and ACAN predicted by the STRING tool. (C) Mouse primary chondrocytes were transfected *Hsp90ab1*-siRNA (SI) for 24 h. The efficiency of silence was measured by qRT-PCR. (D) After continuous injection of siRNA-*Hsp90ab1* in joint cavity for 4 weeks. The efficiency of silence was measured by qRT-PCR. n=3, * p < 0.05, *** p < 0.001. Data are shown as mean ± SEM. P values were obtained by Student’s t tests with two groups.


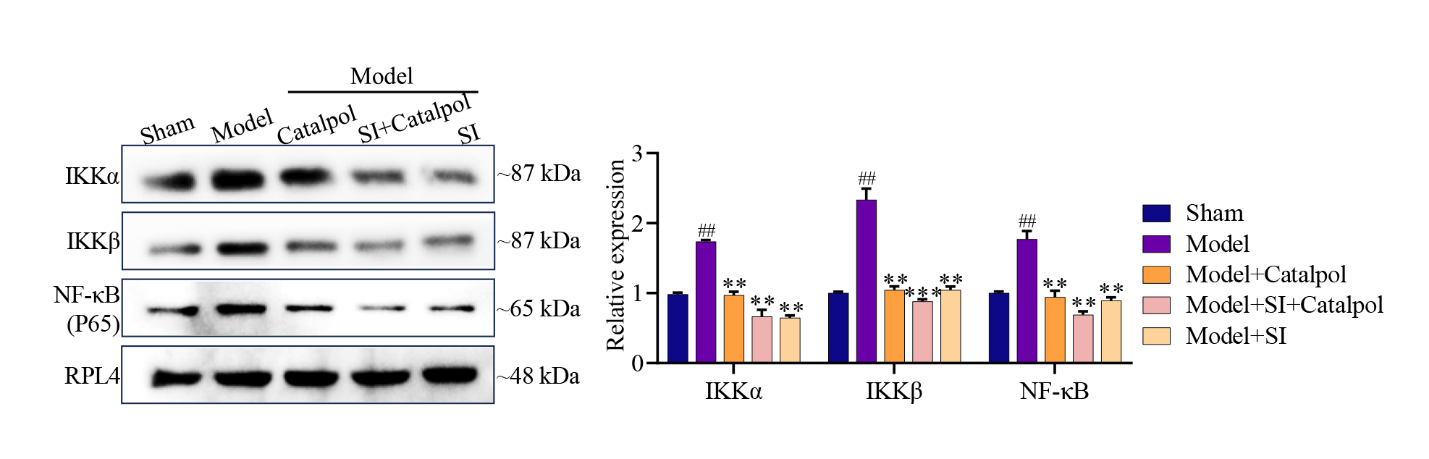


**Figure S12. Hsp90β is required for catalpol regulation NF-κB signaling pathway.** The changes of NF-κB signaling pathway between different groups were detected by using Western blot assay. The quantification result was shown at right. (n = 3, ## or ** p < 0.01, ### or *** p < 0.001 when compared with control group and IL-1β group, respectively). Data are shown as mean ± SEM. P values were obtained by one-way ANOVA with multiple comparisons.

**
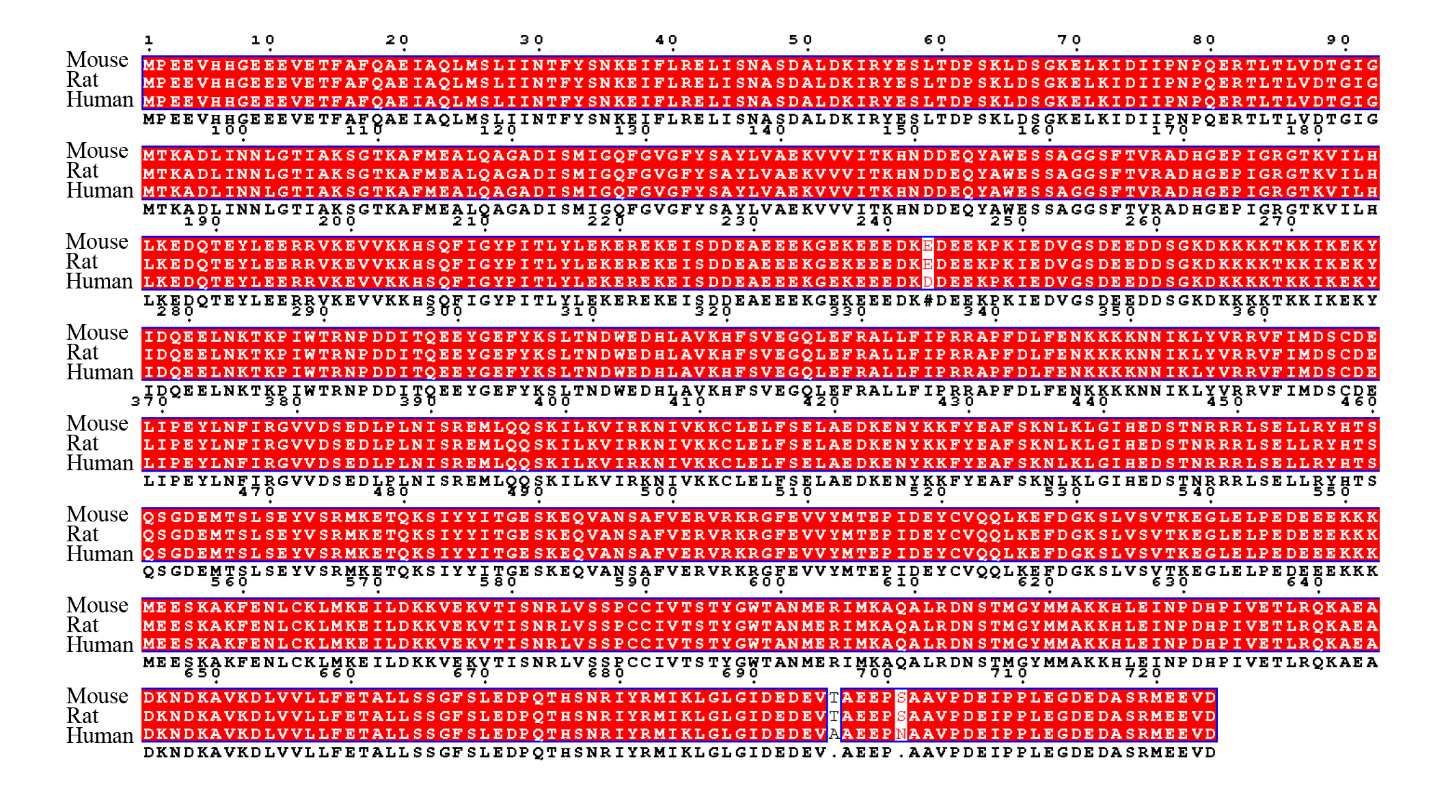
**

**Figure S13. Full length of Hsp90β protein amino acid sequence alignment between mouse, rat and human.**

**Table. S1 Primer Sequence**

| Gene | Primer | Sequence (5’-3’) |
| --- | --- | --- |
| Col2a1-mouse | Forward | GGTCCTCAAGGCAAAGTTGG |
|  | Reverse | TTGCCAGGAAGACCTCTCAG |
| Acan-mouse | Forward | TCTACCTCTACCCCAACCAG |
|  | Reverse | ACGATCCAGTCCTCTATGTCAG |
| Comp-mouse | Forward | ACTGCCTGCGTTCTAGTGC |
|  | Reverse | CGCCGCATTAGTCTCCTGAA |
| Mmp9-mouse | Forward | CCTGGAACTCACACAACGTC |
|  | Reverse | TGCAGGAGGTCATAGGTCAC |
| Mmp13-mouse | Forward | CAGATTCTTCTGGCGTCTGC |
|  | Reverse | CTCGGGATGGATGCTCGTAT |
| Adamts4-mouse | Forward | CAACGTCAAGGCTCCTTCTG |
|  | Reverse | GTTTCGGATGCTTGGATGCT |
| Hsp90ab1-mouse | Forward | GTCCGCCGTGTGTTCATCAT |
|  | Reverse | GCACTTCTTGACGATGTTCTTGC |
| Hsp90ab1-rat | Forward | GCTCCTTCGCTACCATACCT |
|  | Reverse | CACCTGCTCTTTGCTCTCAC |
| Rpl4-mouse | Forward | GTATGGCACTTGGCGTAAGG |
|  | Reverse | AATCTTCTTGCGTGGTGCTC |
| Col2a1-human | Forward | CCAGATGACCTTCCTACGCC |
|  | Reverse | TTCAGGGCAGTGTACGTGAAC |
| Acan-human | Forward | GTGCCTATCAGGACAAGGTCT |
|  | Reverse | GATGCCTTTCACCACGACTTC |
| Mmp13-human | Forward | CCAGACTTCACGATGGCATTG |
|  | Reverse | GGCATCTCCTCCATAATTTGGC |
| Adamts4-human | Forward | GAGGAGGAGATCGTGTTTCCA |
|  | Reverse | CCAGCTCTAGTAGCAGCGTC |
| Rpl4-human | Forward | TGTTTGCACCAACCAAAACCT |
|  | Reverse | GCAGAACAGATGGCGTATCGT |

**Table. S2 SiRNA Sequence**

| Name |  | Sequence (5’-3’) |
| --- | --- | --- |
| Hsp90ab1-mouse | Sense | GAUGAGAUGACCUCCUUGUTT |
|  | Antisense | ACAAGGAGGUCAUCUCAUCTT |
| Si-NC-mouse | Sense | UUCUCCGAACGUGUCACGUTT |
|  | Antisense | ACGUGACACGUUCGGAGAATT |
| Hsp90ab1-rat | Sense | CACAGAAGUCCAUCUACUATT |
|  | Antisense | UAGUAGAUGGACUUCUGUGTT |
| Si-NC-rat | Sense | UUCUCCGAACGUGUCACGUTT |
|  | Antisense | ACGUGACACGUUCGGAGAATT |


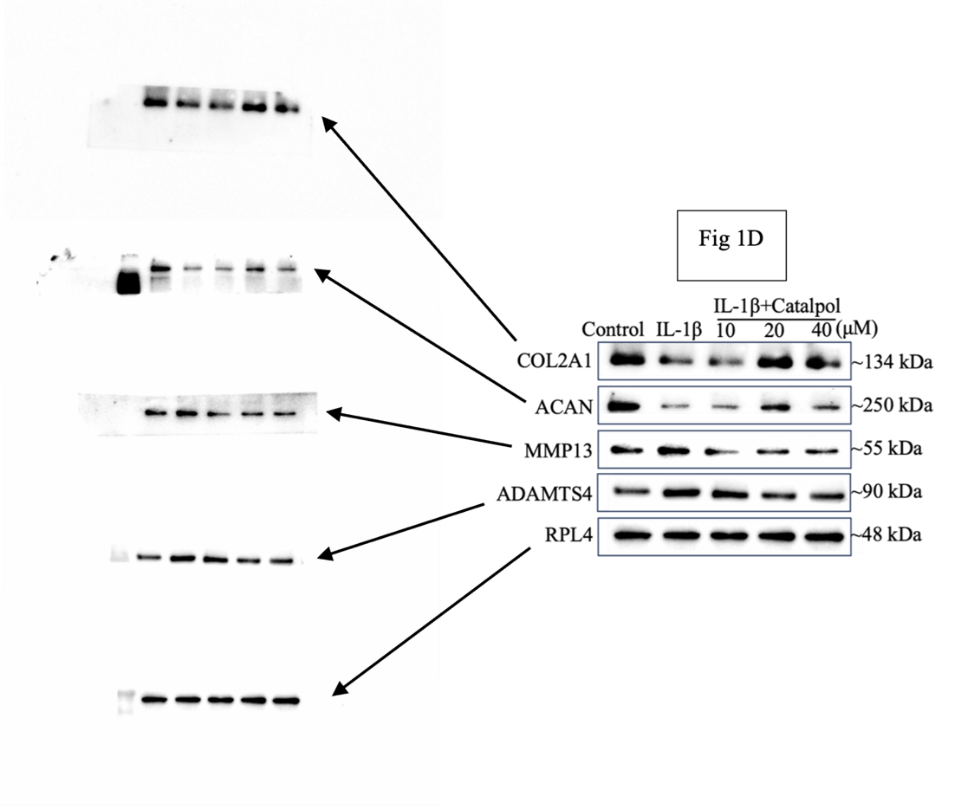


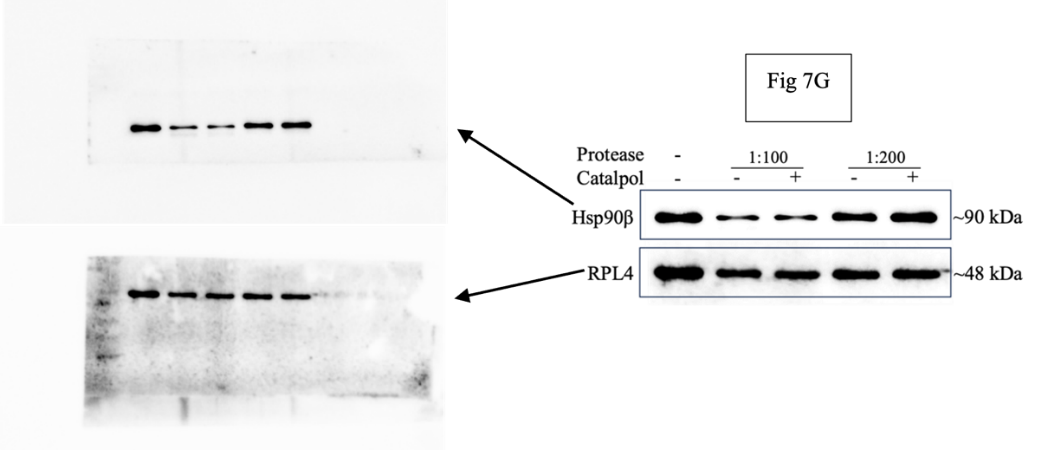


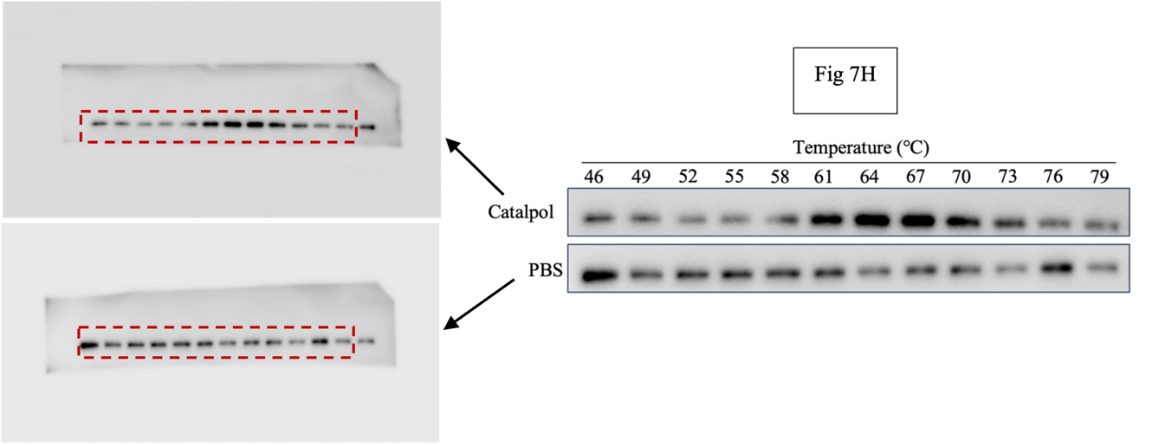


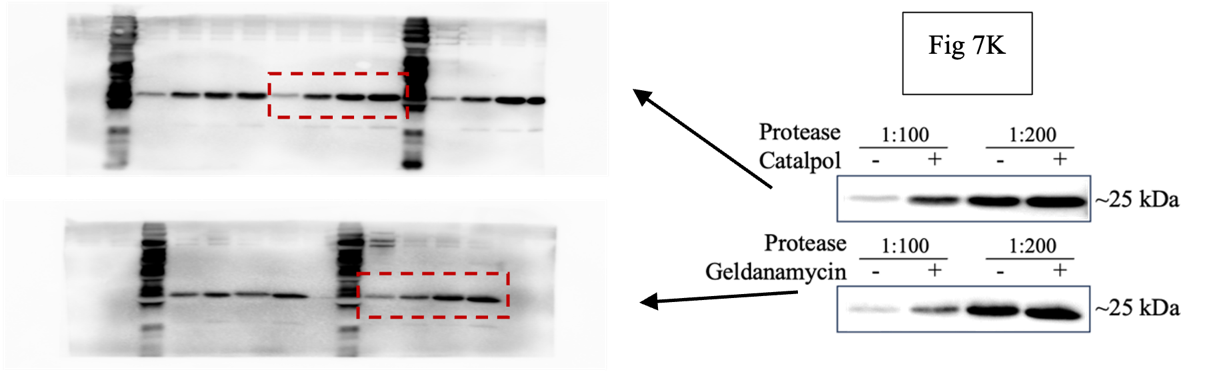


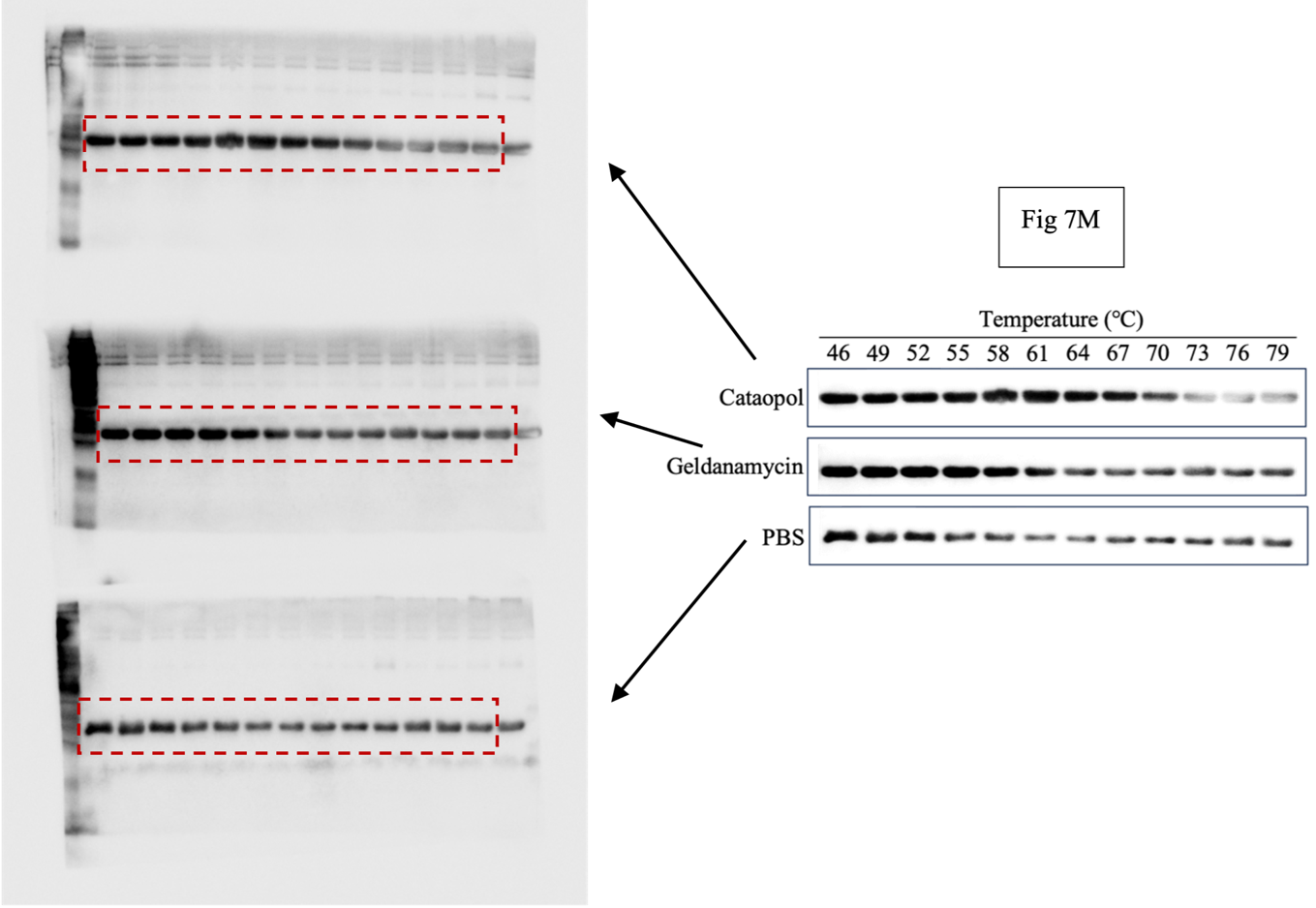


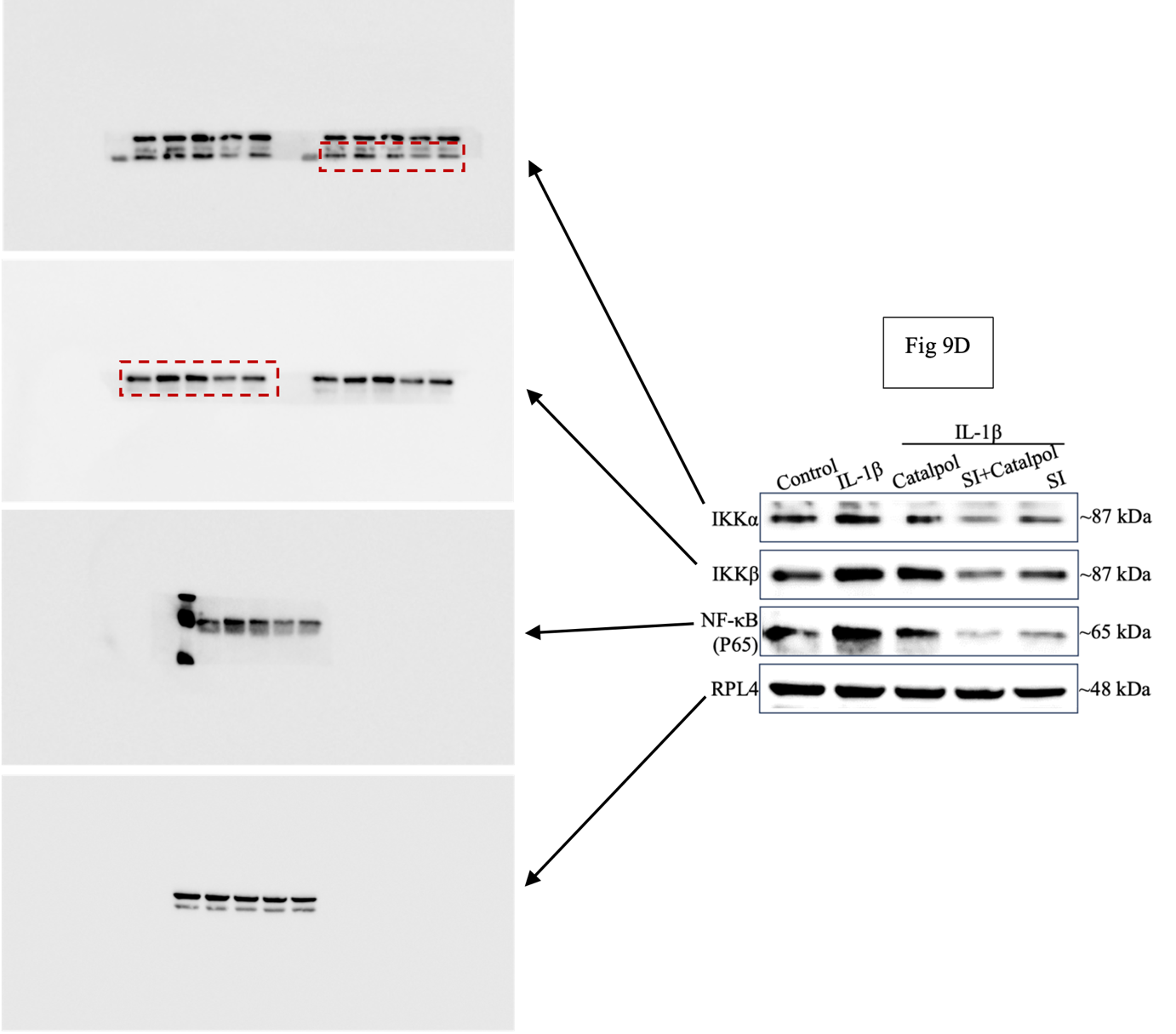


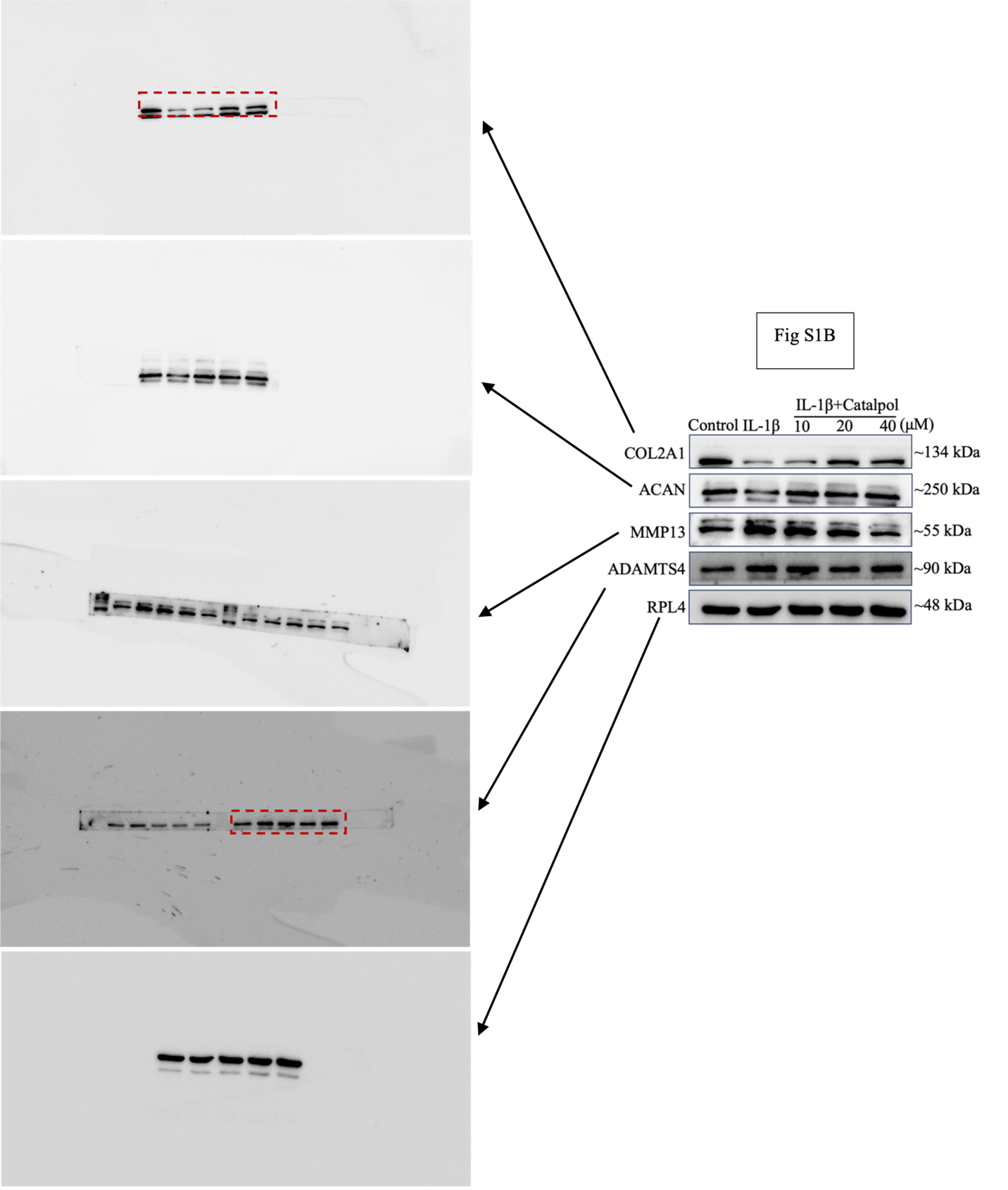


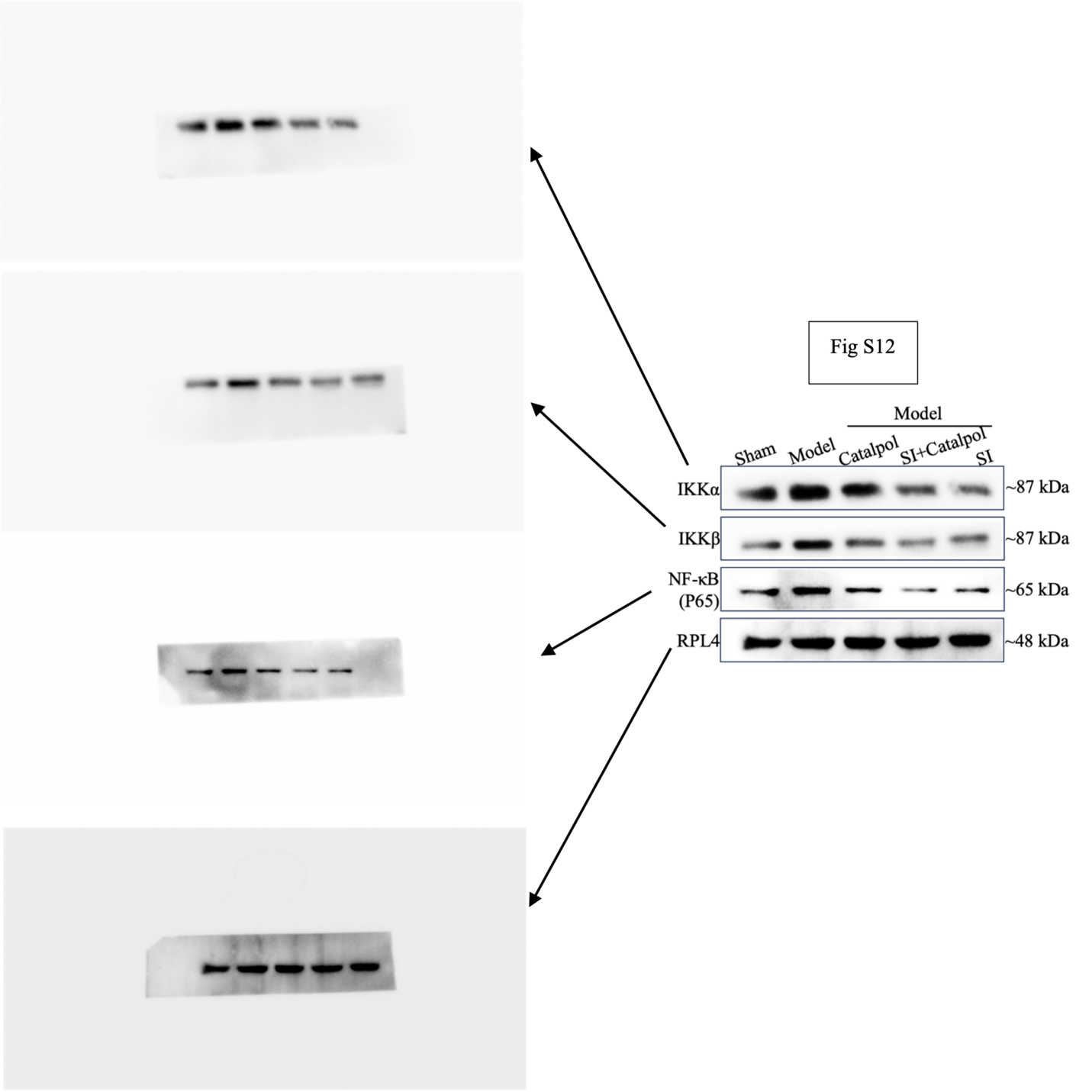

Supplement: Supplementary file 1 — Supporting Information [file ADVS-12-2503909-s001.docx]
